# Supplementary material for: Neonatal azithromycin administration to prevent infant mortality: study protocol for a randomised controlled trial
Source: BMJ Open. 2019 Sep 4;9(9):e031162. doi: 10.1136/bmjopen-2019-031162 (PMC6731835; doi:10.1136/bmjopen-2019-031162)
Supplement: Supplementary data [file bmjopen-2019-031162supp001.pdf]

UNIVERSITY OF CALIFORNIA, SAN FRANCISCO CENTRE DE RECHERCHE EN SANTE DE NOUNA PARENTAL CONSENT TO PARTICIPATE IN A RESEARCH STUDY

This is a medical research study. This study will be explained to you by a staff member from the Centre de Recherche en Santé de Nouna (CRSN). The principal investigator, Dr. Ali Sié from the CRSN, tel: [redacted] and the other principal investigator for this study, Dr Thomas Lietman from the university of California in San Francisco, are available by phone to answer your questions at [redacted].

Medical research studies include only people who choose to take part. Take your time to make your decision about having your child participating in the study. You may discuss your decision with your family and friends. If you have any questions, you may ask your study doctors or the personnel of the CRSN in Nouna.

Why is this study being done?

The purpose of this study is to reduce the childhood mortality during the neonatal period (the first month of life)

How many children will take part in this study?

About 21,000 children will take part in this study.

What will happen if my child takes part in this research study?

If you decide that your child will take part in this study, we will give your child a single oral dose of Azithromycin or placebo between the 8th and the 27th day of life.

Your child might receive Azithromycin, but s/he might also receive a placebo. The placebo will look and taste the same as the azithromycin but will not contain any active medication.

The treatment assignment will be randomly decided by chance, similar to a lottery, with an equal chance of the infant receiving either azithromycin or placebo. The placebo will look and taste the same as Azithromycin but will not contain any active medicine.

After the treatment has been administered we will come to your house 6 times and ask you a few question about your child and his/her health status. For example we will ask you if your child was sick since the last time we saw him/her, we will also ask if your child still lives with you. We will come to your house 7, 14, 28, 90, 180, and 365 days after the treatment day.

Before the treatment administration and when your child is approximatively 6 months old we will measure your child's length, weight and Middle Upper Arm circumference (the size of the contour of your child's arm).

Sie A, *et al. BMJ Open* 2019; 9:e031162. doi: 10.1136/bmjopen-2019-031162

How long will my child be in the study?

The entire time your child will be in the study is approximatively 1 hour and 55 minutes over a period of 1 year.

- The single administration of the treatment will take approximatively 5 minutes.
- Each follow up visit after your child has been treated will take approximatively 15 minutes.
- The anthropometric measurements will take approximatively 10 minutes each time (20 minutes total)

Can my child stop being in the study?

Yes. You can decide to stop at any time. Tell the study doctor or the personnel at the CRSN if you are thinking about stopping or decide to stop. He or she will tell you how to stop your child's participation safely.

What side effects or risks can my child expect from being in the study?

Your child may have side effects due to the treatment.

The most common side effects with this kind of antibiotics are: diarrhea, abdominal pain, vomiting or rash. Every child taking part in this study will be carefully watched for any side effects.

Taking the treatment might also increase the risk of your child developing infantile hypertrophic pyloric stenosis (IHPS). IHPS is when the opening between the stomach and small intestines thickens, if this happens your child will vomit and won't be able to feed properly. The only way to treat IHPS is to perform a surgery that will widen the opening between the stomach and the small intestines. This is an uncommon condition and your child will be monitored very closely for any early signs of IHPS. If your child develops symptoms that might be related to this condition s/he will be seen immediately by a pediatrician for further evaluation.

Your child might also be allergic to the study drug, the most common allergic reactions to this kind of treatment are: rash, itching or dizziness. The serious allergic reactions such as shortness of breath or swelling of the face/tongue/lips are rare and will be monitored closely by our team.

Your child might experience some discomfort during the anthropometric measurements but the risk of hurting your child is minimal. We will take all the precautions to not hurt your child during this procedure and only medical trained staff will perform the measurements.

You should talk to a staff member of the CRSN if your child experiences any side effect.

Sie A, *et al. BMJ Open* 2019; 9:e031162. doi: 10.1136/bmjopen-2019-031162

Are there benefits to taking part in the study?

If your child is suffering from an undiagnosed infectious disease, s/he might directly benefit from the receipt of azithromycin. Not all children will benefit from being in the study and not all the children will receive treatment by being in the study. The children who receive placebo will not receive any benefit or treatment.

In a recent study done in Niger, Tanzania and Malawi, the administration of the azithromycin reduced by 25% the mortality rate in children aged 1 to 6 months.

What other choices do I have if my child does not take part in this study?

You can decide to take part in this study or not take part in this study. Your participation is voluntary. It is your choice whether or not your child will be part of this study.

Nothing will happen to you or your child if you decide that your child won't take part in this study.

How will information about my child be kept confidential?

We will keep all of your child's information confidential. Some entities might be able to access study records, in particular, the University of California at San Francisco Institutional Review Board, the Centre de Recherche en santé de Nouna, and the US Office for Human Research Protections.

There always exists the risk of loss/breach of confidentiality of study data, but that we will take measures to prevent that from happening. We will not use your child's name for anything, except to make sure they are offered treatment when we come back.

Who can answer my questions about the study?

If you have any questions about the study or your rights as a participant to someone other than the researchers or if you have any concerns about the study, please contact the Chair of the Health Research Ethics Board (HREB) Pr. Seni Kouanda 09 P Boîte 7009 Ouagadougou 09, tel: +226 25 36 6674 mobile: +226 70 261462 or the President of the NSRF Institutional Ethics Committee: Mr Zoumbara Jean Désiré, tel: +22670716530, P Box 02, Nouna.

This is a medical research study. This study will be explained to you by a staff member of the Centre de Recherche en Santé de Nouna (CRSN). Dr Ali Sié, NSRF Principal Investigator, telephone: [redacted] is available to answer your questions, Dr Mamadou Bountogo, NSRF Coordinator, telephone:

Sie A, *et al. BMJ Open* 2019; 9:e031162. doi: 10.1136/bmjopen-2019-031162

[redacted] and the other Principal Investigator, Dr Thomas Lietman of the University of California at San Francisco, is also available to answer your questions by telephone: [redacted].

Sie A, *et al. BMJ Open* 2019; 9:e031162. doi: 10.1136/bmjopen-2019-031162

CONSENT

I have read the informed consent form, or it has been read to me. I fully understand the above information. I had the opportunity to ask questions, and I got satisfactory answers. I voluntarily consent for my child to participate in this research. I understand that I have the right to decline to my child's participation or to withdraw at any time without penalty or loss of benefits to which I am otherwise entitled.

____________ ____
Date Participant's Signature or fingerprint *(in case participant is illiterate)*

____________ ____
Date Participant's Witness Signature *(in case participant is illiterate)*

____________ ____
Date Person Obtaining Consent

Sie A, *et al. BMJ Open* 2019; 9:e031162. doi: 10.1136/bmjopen-2019-031162
