# Supplementary material for: Neonatal azithromycin administration to prevent infant mortality: study protocol for a randomised controlled trial
Source: BMJ Open. 2019 Sep 4;9(9):e031162. doi: 10.1136/bmjopen-2019-031162 (PMC6731835; doi:10.1136/bmjopen-2019-031162)
Supplement: Author's manuscript [file bmjopen-2019-031162.draft_revisions.pdf]

BMJ Open

BMJ Open is committed to open peer review. As part of this commitment we make the peer review history of every article we publish publicly available.

When an article is published we post the peer reviewers' comments and the authors' responses online. We also post the versions of the paper that were used during peer review. These are the versions that the peer review comments apply to.

The versions of the paper that follow are the versions that were submitted during the peer review process. They are not the versions of record or the final published versions. They should not be cited or distributed as the published version of this manuscript.

BMJ Open is an open access journal and the full, final, typeset and author-corrected version of record of the manuscript is available on our site with no access controls, subscription charges or pay-per-view fees (http://bmjopen.bmj.com).

If you have any questions on BMJ Open's open peer review process please email editorial.bmjopen@bmj.com

BMJ Open

Neonatal azithromycin administration to prevent infant mortality: design and rationale of a randomized controlled trial

Journal:	*BMJ Open*
Manuscript ID	bmjopen-2019-031162
Article Type:	Protocol
Date Submitted by the Author:	22-Apr-2019

Complete List of Authors:	Sie, Ali; Centre de Recherche en Sante de Nouna<br>Bountogo, Mamadou; Centre de Recherche en Sante de Nouna<br>Nebie, Eric; Centre de Recherche en Sante de Nouna<br>Ouattara, Mamadou; Centre de Recherche en Sante de Nouna<br>Coulibaly, Boubacar; Centre de Recherche en Sante de Nouna<br>Bagagnan, Cheik; Centre de Recherche en Sante de Nouna<br>Zabre, Pascal; Centre de Recherche en Sante de Nouna<br>Lebas, Elodie; University of California San Francisco, Francis I Proctor Foundation<br>Brogdon, Jessica; University of California San Francisco, Francis I Proctor Foundation<br>Godwin, William; University of California San Francisco, Francis I Proctor Foundation<br>Lin, Ying; University of California San Francisco, Francis I Proctor Foundation<br>Porco, Travis; University of California, San Francisco, F.I. Proctor Foundation; University of California, San Francisco, Department of Ophthalmology<br>Doan, Thuy; University of California San Francisco, Francis I Proctor Foundation<br>Lietman, Thomas; University of California, San Francisco<br>Oldenburg, Catherine; University of California San Francisco, Francis I Proctor Foundation

Keywords:	azithromycin, neonatal mortality, sub-Saharan Africa, randomized controlled trial

SCHOLARONE™
Manuscripts

Neonatal azithromycin administration to prevent infant mortality: design and rationale of a randomized controlled trial

Ali Sié[1], Mamadou Bountogo[1], Eric Nebie[1], Mamadou Ouattara[1], Boubacar Coulibaly[1], Cheik Bagagnan[1], Pascal Zabré[1], Elodie Lebas[2], Jessica M. Brogdon[2], William W. Godwin[2], Ying Lin[2], Travis C. Porco[2,3,4], Thuy Doan[2,3], Thomas M. Lietman[2,3,4], Catherine E. Oldenburg[2,3,4]* for the NAITRE Study Group

[1]Centre de Recherche en Santé de Nouna, Nouna, Burkina Faso
[2]Francis I. Proctor Foundation, University of California, San Francisco, USA
[3]Department of Ophthalmology, University of California, San Francisco, USA
[4]Department of Epidemiology and Biostatistics, University of California, San Francisco, USA

***Author for Correspondence:**
Catherine E. Oldenburg, ScD MPH
513 Parnassus Ave, Room S334
San Francisco, CA, USA
Phone: 415-502-8843
Email: catherine.oldenburg@ucsf.edu

Word Count: 3161
Abstract Word Count: 238
Figures: 3
References: 26

For peer review only - http://bmjopen.bmj.com/site/about/guidelines.xhtml

ABSTRACT

Background. Biannual mass azithromycin distribution to children aged 1-59 months has been shown to reduce all-cause mortality. Children under 28 days of age were not treated due to concerns related to infantile hypertrophic pyloric stenosis (IHPS). Here, we report the design of a randomized controlled trial to evaluate the efficacy and safety of administration of a single dose of oral azithromycin during the neonatal period.

Methods and Analysis. The *Nouveaux-nés et Azithromycine: une Innovation dans le Traitement des Enfants* (NAITRE) study is a double-masked randomized placebo-controlled trial designed to evaluate the efficacy of a single dose of azithromycin (20 mg/kg) for the prevention of child mortality. Newborns (*N*=21,712) aged 8 to 27 days weighing at least 2500 g are 1:1 randomized to a single, directly observed, oral dose of azithromycin or matching placebo. Participants are followed weekly for three weeks after treatment to screen for adverse events, including IHPS. The primary outcome is all-cause mortality at the 6-month study visit.

Ethics and Dissemination. This study was approved by the Institutional Review Boards at the University of California, San Francisco in San Francisco, USA (Protocol #18-25027) and the Comité National d'Ethique pour la Recherche in Ouagadougou, Burkina Faso (Protocol #2018-10-123). The findings of this trial will be presented at local, regional, and international meetings and published in open access peer-reviewed journals.

Trial Registration. This study is registered at clinicaltrials.gov (NCT03682653); prospectively registered pre-results.

Key Words. azithromycin; neonatal mortality; sub-Saharan Africa; randomized controlled trial

For peer review only

Strengths and Limitations of this Study

- This study is a large individually randomized placebo-controlled trial evaluating a single dose of azithromycin compared to a single dose of matching placebo during the neonatal period for the prevention of infant mortality.
- This study will use an identical matching placebo, which will minimize bias between the two arms, and participants, outcome assessors, and investigators are masked to treatment allocation.
- Infants will be carefully monitored for adverse events following treatment administration to screen for infantile hypertrophic pyloric stenosis (IHPS) and other potential adverse events.
- Limitations of the study include the limited number of assessments that can be made with a large sample size and that the study may be underpowered to detect IHPS, given the expected rarity of the condition.

For peer review only

INTRODUCTION

The MORDOR study demonstrated that mass azithromycin distribution reduces all-cause child mortality relative to placebo in high mortality settings in sub-Saharan Africa.[1,2] The largest effects were seen in the youngest children, with nearly 25% reduction in mortality in children aged one to five months. Children in this age range have the highest risk of mortality, and thus may have the most to gain from child survival interventions. MORDOR treated children as young as one month of age due to increased mortality in this population, but did not treat children before 28 days of life due to concerns related to potential increased risk of infantile hypertrophic pyloric stenosis (IHPS) related to macrolide use.[3,4] No cases of IHPS or increases in vomiting were found in infants aged 1 to 5 months receiving azithromycin in MORDOR relative to placebo.[5]

Globally, reductions in neonatal mortality have been slower than reduction in post-neonatal mortality, and slow progress in reduction in neonatal mortality has affected overall progress in achieving under-five mortality targets.[6,7] The majority of neonatal deaths occur during the first week of life, a majority of which are due to preterm birth and intrapartum-related conditions.[7,8] Neonatal mortality is more likely to be infectious in nature in the late neonatal period (8-27 days), during which time the most common cause of neonatal mortality is sepsis.[7] Post-neonatal mortality (mortality in children aged 28 to 364 days) is higher than in older children, and in sub-Saharan Africa mortality in this age group is largely infectious in nature.[9] Any effect of azithromycin for prevention of child mortality is likely achieved through reductions in infectious mortality.[10] Distribution of azithromycin during early infancy when the risk of infectious mortality is highest may prove to be an efficacious strategy for targeting azithromycin treatment

For peer review only

for child survival. Here, we describe an individually randomized placebo-controlled double-masked trial designed to establish the efficacy of administration of azithromycin during the neonatal period for the prevention of child mortality. Our central hypothesis is that a single oral dose of azithromycin administered during the neonatal period will reduce all-cause infant mortality, compared to a single dose of placebo.

METHODS/DESIGN

Study design. The *Nouveaux-nés et Azithromycine: une Innovation dans le Traitement des Enfants* (NAITRE; clinicaltrials.gov NCT03682653) study is an individually randomized placebo-controlled trial designed to evaluate the efficacy and safety of administration of a single dose of oral azithromycin during the neonatal period for the prevention of infant mortality (**Figure 1**). Neonates aged 8 to 27 days are randomized in a 1:1 fashion to a single dose of oral azithromycin or matching placebo and followed for 12 months. The primary outcome of the trial is 6-month infant mortality (**Figure 2**). Enrollment commenced in April 2019 and is expected to last until mid-2022.

Objective and hypothesis. The objective of this study is to establish the safety and efficacy of administration of a single dose of oral azithromycin to neonates aged 8 to 27 days for the prevention of infant mortality. We hypothesize that neonates randomized to azithromycin will have significantly lower all-cause mortality by 6 months of age compared to those randomized to placebo.

Study oversight. An independent Data and Safety Monitoring Committee (DSMC) oversees data collection and patient safety in this study. The DSMC contains members with expertise in

For peer review only - http://bmjopen.bmj.com/site/about/guidelines.xhtml

pediatrics, infectious disease, biostatistics, bioethics, and mass azithromycin distribution. The DSMC meets annually in a face-to-face meeting, including once prior to commencement of the study. Quarterly phone meetings during the implementation of the trial take place to monitor progress and review safety data. In addition to the DSMC, a trial steering committee headquartered in Burkina Faso annually reviews the progress of the meeting. The steering committee meets annually to review trial progress and consists of key stakeholders in policy, child health, and research in Burkina Faso. Serious adverse events are reviewed by two medical monitors, one based in Burkina Faso and one in the United States.

Setting. This study is taking place in 9 regions throughout Burkina Faso (**Figure 3**). All study sites are within a four-hour drive of a facility with capability of performing pyloromyotomy (Centre Hospitalier Universitaire Pédiatrique Charles de Gaulle in Ouagadougou or Centre Hospitalier Universitaire Souro Sanou in Bobo Dioulasso). Study sites are situated in peri-urban (e.g., outside of Ouagadougou) or rural areas. Pregnancies, births, and newborns will be identified and enrolled at a *Centre de Santé et de Promotion Sociale* (CSPS) in the study area. CSPSs are government nurse-led primary care facilities that offer preventive and curative care to catchment areas covering several rural villages.[11] Study headquarters are at the Centre de Recherche en Santé de Nouna in Nouna, northwestern Burkina Faso.

Recruitment. Newborns are recruited through several mechanisms, including identification of pregnant women in their third trimester, facility-based births, and key informant or community health worker systems in study areas. Pregnant women are informed about the study and asked to contact study staff after delivery if they are interested in their child participating in the trial.

For peer review only - http://bmjopen.bmj.com/site/about/guidelines.xhtml

Women who give birth in facilities participating in the study are approached prior to discharge about the study and asked to return with their infant in 1 to 2 weeks for eligibility assessment and enrollment. Verbal consent is obtained from pregnant women and women who have recently delivered to collect contact information, follow their pregnancy, and contact them when the child is in the eligible window for enrollment if they are interested in their child participating in the study. Caregivers and newborns are also referred to the study by community health workers and key informants who work in the communities in the catchment areas for participating study clinics.

Inclusion and exclusion criteria. Newborns are eligible to be enrolled if they are from 8 to 27 days of age, weigh at least 2500 g at the time of enrollment, have no known allergy to azalides, no hepatic failure as indicated by neonatal jaundice, and are able to feed orally. In addition, the family of the newborn must intend to stay in the study area for at least 6 months and appropriate consent must be obtained from the caregiver.

Enrollment and baseline survey. After screening and prior to formal enrollment, written informed consent is obtained from the caregiver of all participants. Participants are then enrolled into the study and assigned a study identification number. The baseline assessment includes anthropometry (weight, length, and mid-upper arm circumference measurement), child's birthweight (obtained from the child's health card), timing of initiation of breastfeeding, and pregnancy type (singleton or multiple).

For peer review only - http://bmjopen.bmj.com/site/about/guidelines.xhtml

Randomization. Children are randomized in a 1:1 fashion to a single dose of azithromycin or placebo. The randomization list was generated by TCP and WWG in R version 3.3. Treatment letters that correspond to azithromycin or placebo were randomly assigned to study identification numbers. Children are assigned via the study's mobile application to a study identification number and thus a randomization letter. To prevent accidental unmasking, a total of 8 letters are used in the study, with four referring to azithromycin and four referring to placebo. Treatment bottles are labeled with study-specific labels that are identical in appearance with the exception of the treatment letter to facilitate masking.

Treatment and masking. Treatment is provided as a single oral directly observed dose of azithromycin (approximating 20 mg/kg, weight-based dosing) or matching placebo. Azithromycin for oral suspension is provided in bottles containing azithromycin dehydrate powder equivalent to 1200 mg per bottle. The placebo oral suspension is identical to the azithromycin with the exception of the active ingredient to achieve masking. After children are weighed as part of the baseline anthropometric assessment, the tablet will automatically calculate the dosage of treatment. All study medications, including azithromycin and matching placebo, are donated by Pfizer, Inc.

Allocation concealment. Allocation concealment is achieved via masking and the electronic tablet. With a fully-masked trial, study staff, investigators, and participants are not aware of which assignment they will be given or have received. Furthermore, study staff are not aware of the randomization letters that will be assigned to the next participant until that participant has undergone screening, informed consent, baseline assessment, and randomization. After the

For peer review only

For peer review only - http://bmjopen.bmj.com/site/about/guidelines.xhtml

baseline visit is complete, the tablet displays the treatment letters corresponding to the medication bottle with which the child will be treated. Viewing treatment letters prior to completion of the baseline form is not possible on the tablet, thus achieving allocation concealment.

Follow-up. All participants complete follow-up visits weekly for three weeks after treatment, and then at 3, 6, and 12 months of age. The weekly adverse event follow-up visits and the 3- and 12-month visits occur via phone call or home visit. The 6-month visit is an in-person visit conducted at the CSPS.

Primary outcome measurement. The primary outcome is all-cause mortality determined at the 6-month study visit. Vital status is measured via interview with the caregiver or head of household. Children who are living will be evaluated in person at the CSPS or via home visit. Vital status will be recorded in the study's mobile application via tablet.

Secondary outcome measurements.

12-month mortality. Vital status will be measured via caregiver interview determined at 12 months of age. The 12-month study visit will be conducted via phone call or in-person visit.

Neonatal mortality. As a pre-specified secondary outcome, we will assess neonatal mortality (mortality prior to 28 days of age) by study arm. Vital status will be measured in all children at each weekly follow-up visit for three weeks, and as such 28-day mortality will be measured in all children regardless of age of enrollment. Although we do not anticipate that this secondary

For peer review only - http://bmjopen.bmj.com/site/about/guidelines.xhtml

analysis will be fully powered, it is pre-specified to assess if azithromycin dosing during the neonatal period has a large effect on neonatal mortality.

Hospitalization. At each study visit, interviewers will be asked if they had sought medical care for their child since they last spoke with the study team, and if so if their child had stayed overnight in a healthcare facility.

Anthropometric outcomes. At the 6-month study visit, anthropometric measurements including weight, length, and mid-upper arm circumference are collected from all children. Weight gain from baseline in grams per kilogram per day (g/kg/day) will be calculated to estimate weight gain velocity by study arm. Length gain in mm/day will be calculated by study arm. Weight-for-height Z-score, height-for-age Z-score, and weight-for-age Z-score will be calculated according to 2006 World Health Organization (WHO) standards.[12]

Cause-specific mortality. A verbal autopsy interview will be undertaken with the caregiver of all children who die during the course of the study using the 2014 WHO verbal autopsy instrument or the neonatal verbal autopsy instrument if the death occurs before 4 weeks of age. Cause of death data will be analyzed using the InterVA algorithm.[13,14]

Adverse events. The primary adverse event under surveillance is IHPS. Active surveillance for IHPS occurs via weekly screening of all enrolled children for three weeks after treatment. In addition, during the informed consent process the caregiver of all enrolled children is informed of the signs and symptoms of IHPS and asked to contact study staff should the child exhibit

For peer review only - http://bmjopen.bmj.com/site/about/guidelines.xhtml

abnormal vomiting. All caregivers are given a fact sheet with information about IHPS and its signs and symptoms. If active or passive surveillance identifies a child with projectile or progressive vomiting that occurs consistently after eating (i.e., the child is unable to eat anything without vomiting), the child will be referred to the district hospital for evaluation of clinical symptoms and possible referral to the regional or national hospital for an ultrasound. If the ultrasound indicates that the pylorus is hypertrophied per Burkinabé national guidelines (>4 mm for pyloric muscle thickness or >15 mm for pyloric length) or the measurements are within normal limits but no food can pass per dynamic ultrasound, the child will be transferred to a national hospital for further evaluation, diagnosis, and pyloromyotomy if IHPS is diagnosed. Children diagnosed with IHPS will be followed at one week and four weeks after surgery to ascertain surgical outcomes, and then according to the study schedule.

In addition to screening for vomiting and IHPS, caregivers are interviewed weekly for three weeks following treatment for adverse events including rash, diarrhea, and fever. Caregivers are asked if they sought medical care for their child since the last visit by the study team and if so the indication for seeking treatment.

Interim analysis. One pre-specified interim analysis for the primary outcome is planned when full data are available for one-third of the enrollments or after one full year of enrollment (e.g., 6 months after the last individual), whichever comes first. A *P*-value of <0.001 is the pre-specified stopping rule for efficacy for this interim analysis. Thus the final analysis will be conducted at an alpha of 0.049.

For peer review only

Statistical analysis. The primary analysis will be conducted as a logistic regression model, with inferences based on a Monte Carlo permutation test based on the randomization unit. As a pre-specified subgroup analysis, we will assess effects of age at time of treatment in days using a logistic regression model with an interaction for treatment arm by age. All statistical tests for efficacy will be two-sided.

Sample size considerations. Assuming a mortality probability of 35 per 1,000 and loss to follow-up of 10%, a sample size of 10,856 per arm (21,712 total) would yield approximately 80% power to detect a 20% decrease in mortality in children randomized to azithromycin compared to placebo.

Data management. Data are collected via smartphone in the field using Survey Solutions (World Bank Group, Washington, DC), a cloud-based platform for electronic data collection. Smartphones are synced in the field to a cloud-based server.

Patient and public involvement. Members of the community, including key informants in the study communities, are involved in identification of births, recruitment of children, and facilitating follow-up visits. Results will be disseminated in collaboration with community leadership in study areas.

Dissemination plan. Efficacy and safety results of this study will be disseminated to community, policy, and scientific stakeholders, including the Ministry of Health in Burkina Faso, the World Health Organization, and relevant non-governmental organizations that implement

For peer review only - http://bmjopen.bmj.com/site/about/guidelines.xhtml

child health policies. Results will be shared with the scientific community via publication in peer-reviewed journals and presentation at international conferences.

DISCUSSION

Although biannual mass azithromycin distribution has been shown to reduce all-cause child mortality in children aged 1-59 months[1,2], this strategy may not reach the youngest children who may benefit the most. With biannual community distributions, children would first be reached any time between the age of 1 to 7 months, or on average at approximately 4 months of age. Several strategies exist for reliably reaching younger children. For example, quarterly mass treatment would reach children at a younger age on average. Targeted treatment of young children is a strategy that may integrate well with existing health systems and can allow for reliably treated children at specific ages. In Burkina Faso, healthcare for children under 5 is free of charge, and as a result healthcare coverage for children is high. For example, vaccination coverage reportedly exceeded 80% in 2014 in Nouna.[15] If shown to be both efficacious and safe, targeting treatment to the youngest, highest-risk children could be considered via integration with existing well-child visits in the health system.

IHPS is a rare but serious condition, with approximately 2 cases per 1,000 infants in the United States.[4] Surgical treatment is required. However, in many regions of sub-Saharan Africa, infant mortality rates far exceed the risk of IHPS.[24] Prevention of a portion of infant mortality in the highest risk areas may therefore offer substantial benefits for populations. However, the associated risks of IHPS as well as the efficacy must be well understood before any such policy could be put into place. In this study, a comprehensive screening and referral protocol is in place

For peer review only - http://bmjopen.bmj.com/site/about/guidelines.xhtml

to identify and cases of IHPS that arise during the course of the study. We expect that the results of this study will provide evidence of the risks and benefits of treatment of neonates with azithromycin for prevention of child mortality.

To date, existing evidence for the relationship between macrolide use and development of IHPS has been limited to observational studies or small randomized trials of azithromycin for prevention of bronchopulmonary dysplasia in low birthweight infants.[16–18] In a large retrospective cohort study in the United States, among 4,875 infants up to 90 days of life, 8 cases of IHPS occurred, with a higher risk of IHPS among children receiving erythromycin or azithromycin compared to cephalexin.[4] This risk appeared to be higher in children receiving erythromycin compared to azithromycin, and among children under 14 days of age compared to older children. Observational studies are limited by confounding by indication, which could occur if children with conditions necessitating a macrolide have different conditions or are otherwise substantively different from those who receive another antibiotic class or no antibiotics. Existing randomized controlled trials have been small (total N=263 infants randomized), and no cases of IHPS have been detected. Relatively little evidence of the epidemiology of IHPS in sub-Saharan Africa exists[19–21], although some have hypothesized that IHPS is less common in developing country settings compared to high-income settings due to different feeding and medication practices.[21–23] The results of this study are expected to provide comprehensive, rigorous evidence of the relationship between azithromycin use and IHPS in neonates, as well as important data on the epidemiology of IHPS in rural West Africa.

For peer review only - http://bmjopen.bmj.com/site/about/guidelines.xhtml

Several limitations must be considered in this study. This study was designed under the principle of a large simple trial, due to the large sample size required to be adequately powered for a rare outcome.[25,26] However, careful attention must be paid to each individual in the trial due to the risk of IHPS. The focus is on measuring the most important outcomes (mortality and IHPS), and thus measurement of additional secondary outcomes are limited. Furthermore, given the absence of IHPS data from rural sub-Saharan Africa and the expected rarity of the event, the study may be underpowered to detect differences in IHPS between study arms. Stopping rules for safety are based not only on statistical significance, but decisions will be made in conjunction with the DSMC based on case reports arising from the study. We anticipate that the study will provide the largest sample to date providing evidence of the epidemiology of IHPS in West Africa, as well as the largest trial to date evaluating azithromycin for IHPS.

CONCLUSIONS

The results of this study are expected to inform policy related to the use of azithromycin for prevention of child mortality by specifically evaluating the role of targeting doses during early infancy when the risk of infectious mortality is highest. This work is anticipated to build on the evidence base arising from the MORDOR study, which demonstrated that biannual mass treatment significantly reduces all-cause child mortality in children aged 1-59 months. If proven to be safe and effective, the evidence arising from this study could be used to develop policies related to neonatal dosing of azithromycin during the neonatal period.

For peer review only

For peer review only - http://bmjopen.bmj.com/site/about/guidelines.xhtml

FIGURE LEGENDS

Figure 1. Study flow diagram for study visits and outcome assessments.

Figure 2. SPIRIT schedule of enrollment, interventions, and assessments.

Figure 3. Map of study enrollment locations. Red stars indicate enrollment sites. Blue stars indicate sites with pediatric surgical facilities.

For peer review only

For peer review only - http://bmjopen.bmj.com/site/about/guidelines.xhtml

Contributors. Conceived and designed study: AS, MB, EN, MO, BC, CB, PZ, EL, JMB, WWG, YL, TCP, TD, TML, CEO. Created tables/figures: AS, EL, JMB, CEO. Wrote first draft of manuscript: CEO. Read and critically revised manuscript: AS, MB, EN, MO, BC, CB, PZ, EL, JMB, WWG, YL, TCP, TD, TML, CEO. Members of the NAITRE Study Group are listed in the Supplementary File.

Availability of Data and Materials. Upon completion of the trial, de-identified data will be made available in accordance with Bill and Melinda Gates Foundation policies.

Funding. The NAITRE Study is funded by the Bill and Melinda Gates Foundation (OPP1187628). C.E.O and T.D. were supported in part by a Research to Prevention Blindness Career Development Award. The funders played no role in the design, writing, or decision to publish study protocol.

Competing interests. None declared.

Ethical approval. Ethical approval was obtained for the NAITRE Study from the Institutional Review Boards at the University of California, San Francisco (Protocol #18-25027) and the Comité National d'Ethique pour la Recherche (National Ethics Committee of Burkina Faso; Protocol #2018-10-123).

For peer review only

For peer review only - http://bmjopen.bmj.com/site/about/guidelines.xhtml

REFERENCES

1. Keenan JD, Bailey RL, West SK, et al. Mass azithromycin distribution for reducing childhood mortality in sub-Saharan Africa. *N Engl J Med*. 2018;378(17):1583-1592. papers3://publication/uuid/94889A59-49DD-4FAD-BDC0-E1A6B125E22C.
2. Oldenburg CE, Arzika AM, Amza A, et al. Mass azithromycin distribution to prevent childhood mortality: a pooled analysis of cluster randomized trials. *Am J Trop Med Hyg*. 2018;In press:2018.
3. Lund M, Pasternak B, Davidsen RB, et al. Use of macrolides in mother and child and risk of infantile hypertrophic pyloric stenosis: nationwide cohort study. *BMJ*. 2014;348(mar11 8):g1908-g1908. http://www.bmj.com/cgi/doi/10.1136/bmj.g1908.
4. Eberly MD, Eide MB, Thompson JL, Nylund CM. Azithromycin in Early Infancy and Pyloric Stenosis. *Pediatrics*. 2015;135(3):483-488. http://pediatrics.aappublications.org/cgi/doi/10.1542/peds.2014-2026.
5. Oldenburg CE, Arzika AM, Maliki R, et al. Safety of azithromycin in infants under six months of age in Niger : A community randomized trial. *PLoS Negl Trop Dis*. 2018;12(11):e0006950. doi:10.7910/DVN/MQYM5S.Funding
6. Jin Y, Mankadi PM, Rigotti JI. Cause-specific child mortality performance and contributions to all-cause child mortality , and number of child lives saved during the Millennium Development Goals era : a country-level analysis. *Glob Health Action*. 2018;11:1546095. doi:10.1080/16549716.2018.1546095
7. Lawn JE, Blencowe H, Oza S, et al. Every Newborn: progress, priorities, and potential beyond survival. *Lancet*. 2015;384(9938):189-205. doi:10.1016/S0140-6736(14)60496-7
8. Belizan JM, McClure EM, Goudar SS, et al. Neonatal death in low-middle income countries: a global network study. *Am J Perinatol*. 2015;29(8):649-656. doi:10.1055/s-0032-1314885.Neonatal
9. Wang H, Bhutta ZA, Coates MM, et al. Global, regional, national, and selected subnational levels of stillbirths, neonatal, infant, and under-5 mortality, 1980–2015: a systematic analysis for the Global Burden of Disease Study 2015. *Lancet*. 2016;388(10053):1725-1774. http://dx.doi.org/10.1016/S0140-6736(16)31575-6.
10. OBrien KS, Cotter SY, Amza A, et al. Childhood Mortality After Mass Distribution of Azithromycin. *Pediatr Infect Dis J*. 2018;37(11):1082-1086. http://insights.ovid.com/crossref?an=00006454-900000000-96705.
11. Koulidiati J-L, Nesbitt RC, Ouedraogo N, et al. Measuring effective coverage of curative child health services in rural Burkina Faso: a cross-sectional study. *BMJ Open*. 2018;8(5):e020423-10. http://bmjopen.bmj.com/lookup/doi/10.1136/bmjopen-2017-020423.
12. WHO Multicentre Growth Reference Study Group. WHO Child Growth Standards: Length/height-for-age, weight-for-age, weight-for-length, weight-for-height and body mass index-for-age: Methods and development. *Geneva World Heal Organ*. 2006:312.
13. Byass P, Herbst K, Fottrell E, et al. Comparing verbal autopsy cause of death findings as determined by physician coding and probabilistic modeling: a public health analysis of 54 000 deaths in Africa and Asia. *J Glob Health*. 2015;5:1-9. papers3://publication/doi/10.7189/jogh.05.010402.
14. Yé M, Diboulo E, Niamba L, et al. An improved method for physician-certified verbal autopsy reduces the rate of discrepancy: experiences in the Nouna Health and

For peer review only

Demographic Surveillance Site (NHDSS), Burkina Faso. *Popul Health Metr*. 2011;9(1):34. http://www.pophealthmetrics.com/content/9/1/34.
15. Kagoné M, Yé M, Nébié E, et al. Vaccination coverage and factors associated with adherence to the vaccination schedule in young children of a rural area in Burkina Faso. *Glob Health Action*. 2017;10(1):1399749. doi:10.1080/16549716.2017.1399749
16. Smith C, Egunsola O, Choonara I, Kotecha S, Jacqz-Aigrain E, Sammons H. Use and safety of azithromycin in neonates: a systematic review. *BMJ Open*. 2015;5(12):e008194-8. http://bmjopen.bmj.com/lookup/doi/10.1136/bmjopen-2015-008194.
17. Ballard HO, Anstead MI, Shook LA. Azithromycin in the extremely low birth weight infant for the prevention of Bronchopulmonary Dysplasia: a pilot study. *Respir Res*. 2007;8(1):1793-1799. http://respiratory-research.biomedcentral.com/articles/10.1186/1465-9921-8-41.
18. Ballard HO, Shook LA, Bernard P, et al. Use of azithromycin for the prevention of bronchopulmonary dysplasia in preterm infants: a randomized, double-blind, placebo controlled trial. *Pediatr Pulmonol*. 2010;46(2):111-118. http://doi.wiley.com/10.1002/ppul.21352.
19. Chalya PL, Manyama M, Kayange NM, Mabula JB, Massenga A. Infantile hypertrophic pyloric stenosis at a tertiary care hospital in Tanzania: a surgical experience with 102 patients over a 5-year period. *BMC Res Notes*. November 2015:1-6. papers3://publication/doi/10.1186/s13104-015-1660-4.
20. Tadesse A, Gadisa A. Infantile hypertrophic pyloric stenosis: A retrospective study from a tertiary hospital in Ethiopia. *East Cent Afr J Surg*. 2014;19(1):120-124. https://www.ajol.info/index.php/ecajs/article/view/102528/92798.
21. Osifo DO, Evbuomwan I. Does Exclusive Breastfeeding Confer Protection Against Infantile Hypertrophic Pyloric Stenosis? A 30-year Experience in Benin City, Nigeria. *J Trop Pediatr*. 2008;55(2):132-134. https://academic.oup.com/tropej/article-lookup/doi/10.1093/tropej/fmn094.
22. Zhu J, Zhu T, Lin Z, Qu Y, Mu D. Perinatal risk factors for infantile hypertrophic pyloric stenosis: A meta-analysis. *J Pediatr Surg*. 2017;52(9):1389-1397. http://dx.doi.org/10.1016/j.jpedsurg.2017.02.017.
23. Wayne C, Hung J-HC, Chan E, Sedgwick I, Bass J, Nasr A. Formula-feeding and hypertrophic pyloric stenosis: is there an association? A case–control study. *J Pediatr Surg*. 2016;51(5):779-782. http://dx.doi.org/10.1016/j.jpedsurg.2016.02.021.
24. Golding N, Burstein R, Longbottom J, et al. Mapping under-5 and neonatal mortality in Africa, 2000–15: a baseline analysis for the Sustainable Development Goals. *Lancet*. 2017;390(10108):2171-2182. http://dx.doi.org/10.1016/S0140-6736(17)31758-0.
25. Yusuf S, Collins R, Peto R. Why do we need some large, simple trials? *Stat Med*. 1984;3:409-420.
26. Peto R, Collins R, Gray R. Large-scale randomized evidence: large, simple trials and overviews of trials. *J Clin Epidemiol*. 1995;48(1):23-40.

For peer review only - http://bmjopen.bmj.com/site/about/guidelines.xhtml

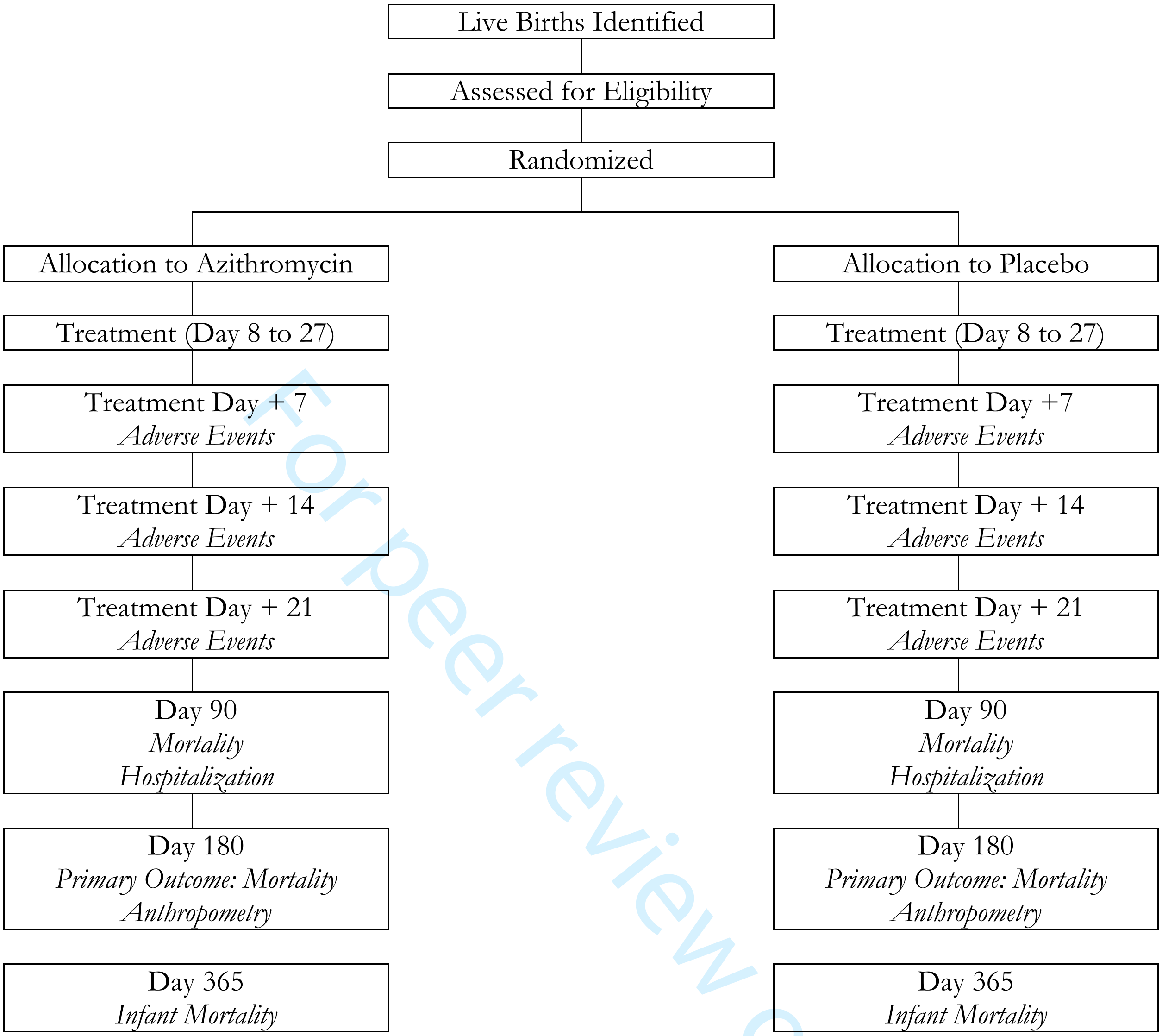

For peer review only

For peer review only - http://bmjopen.bmj.com/site/about/guidelines.xhtml

	STUDY PERIOD					
	Enrolment	Allocation	Post-allocation			Close-out
**TIMEPOINT**		*0*	*Weeks 1, 2, 3*	*3 months*	*6 months*	*12 months*
**ENROLMENT:**						
**Eligibility screen**	X					
**Informed consent**	X					
**Allocation**		X				
**INTERVENTIONS:**						
**Treatment (oral azithromycin or placebo)**		X				
**ASSESSMENTS:**						
**Baseline questionnaire**	X					
**Anthropometry**	X				X	
**Adverse events**			X	X		
**Vital status**			X	X	X	X

For peer review only

For peer review only - http://bmjopen.bmj.com/site/about/guidelines.xhtml

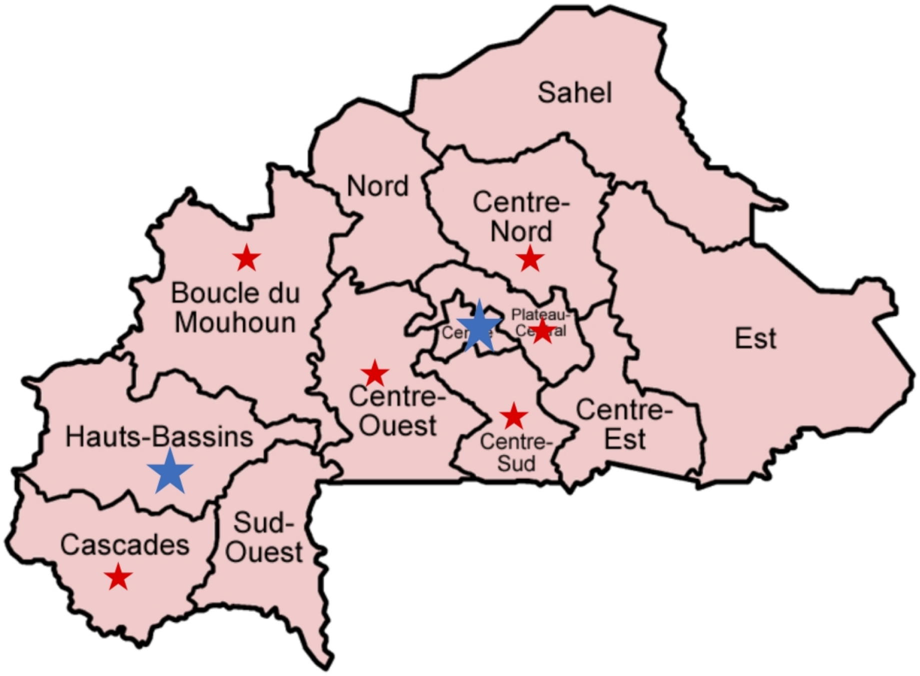

Figure 3

197x140mm (300 x 300 DPI)

SPIRIT
STANDARD PROTOCOL ITEMS: RECOMMENDATIONS FOR INTERVENTIONAL TRIALS

SPIRIT 2013 Checklist: Recommended items to address in a clinical trial protocol and related documents*

Section/item	Item No	Description	Addressed on page number
**Administrative information**			
Title	1	Descriptive title identifying the study design, population, interventions, and, if applicable, trial acronym	________1______
Trial registration	2a	Trial identifier and registry name. If not yet registered, name of intended registry	________2______
	2b	All items from the World Health Organization Trial Registration Data Set	______________
Protocol version	3	Date and version identifier	______n/a______
Funding	4	Sources and types of financial, material, and other support	________17______
Roles and responsibilities	5a	Names, affiliations, and roles of protocol contributors	________1, 17____
	5b	Name and contact information for the trial sponsor	________17______
	5c	Role of study sponsor and funders, if any, in study design; collection, management, analysis, and interpretation of data; writing of the report; and the decision to submit the report for publication, including whether they will have ultimate authority over any of these activities	_________17_____
	5d	Composition, roles, and responsibilities of the coordinating centre, steering committee, endpoint adjudication committee, data management team, and other individuals or groups overseeing the trial, if applicable (see Item 21a for data monitoring committee)	______5______

For peer review only

**Introduction**			
Background and rationale	6a	Description of research question and justification for undertaking the trial, including summary of relevant studies (published and unpublished) examining benefits and harms for each intervention	_____3-4_______
	6b	Explanation for choice of comparators	____4_________
Objectives	7	Specific objectives or hypotheses	_____4________
Trial design	8	Description of trial design including type of trial (eg, parallel group, crossover, factorial, single group), allocation ratio, and framework (eg, superiority, equivalence, noninferiority, exploratory)	_____3-4_______
**Methods: Participants, interventions, and outcomes**			
Study setting	9	Description of study settings (eg, community clinic, academic hospital) and list of countries where data will be collected. Reference to where list of study sites can be obtained	______5_______
Eligibility criteria	10	Inclusion and exclusion criteria for participants. If applicable, eligibility criteria for study centres and individuals who will perform the interventions (eg, surgeons, psychotherapists)	_____6________
Interventions	11a	Interventions for each group with sufficient detail to allow replication, including how and when they will be administered	_____7_____
	11b	Criteria for discontinuing or modifying allocated interventions for a given trial participant (eg, drug dose change in response to harms, participant request, or improving/worsening disease)	_____n/a_______
	11c	Strategies to improve adherence to intervention protocols, and any procedures for monitoring adherence (eg, drug tablet return, laboratory tests)	______n/a______
	11d	Relevant concomitant care and interventions that are permitted or prohibited during the trial	_____n/a______
Outcomes	12	Primary, secondary, and other outcomes, including the specific measurement variable (eg, systolic blood pressure), analysis metric (eg, change from baseline, final value, time to event), method of aggregation (eg, median, proportion), and time point for each outcome. Explanation of the clinical relevance of chosen efficacy and harm outcomes is strongly recommended	___8-9_______

For peer review only - http://bmjopen.bmj.com/site/about/guidelines.xhtml

Participant timeline	13	Time schedule of enrolment, interventions (including any run-ins and washouts), assessments, and visits for participants. A schematic diagram is highly recommended (see Figure)	__8; Figure 2____
Sample size	14	Estimated number of participants needed to achieve study objectives and how it was determined, including clinical and statistical assumptions supporting any sample size calculations	___11__________
Recruitment	15	Strategies for achieving adequate participant enrolment to reach target sample size	___6__________

Methods: Assignment of interventions (for controlled trials)

Allocation:

Sequence generation	16a	Method of generating the allocation sequence (eg, computer-generated random numbers), and list of any factors for stratification. To reduce predictability of a random sequence, details of any planned restriction (eg, blocking) should be provided in a separate document that is unavailable to those who enrol participants or assign interventions	___7__________
Allocation concealment mechanism	16b	Mechanism of implementing the allocation sequence (eg, central telephone; sequentially numbered, opaque, sealed envelopes), describing any steps to conceal the sequence until interventions are assigned	___8__________
Implementation	16c	Who will generate the allocation sequence, who will enrol participants, and who will assign participants to interventions	___7-8________
Blinding (masking)	17a	Who will be blinded after assignment to interventions (eg, trial participants, care providers, outcome assessors, data analysts), and how	___7________
	17b	If blinded, circumstances under which unblinding is permissible, and procedure for revealing a participant's allocated intervention during the trial	__7_________

Methods: Data collection, management, and analysis

Data collection methods	18a	Plans for assessment and collection of outcome, baseline, and other trial data, including any related processes to promote data quality (eg, duplicate measurements, training of assessors) and a description of study instruments (eg, questionnaires, laboratory tests) along with their reliability and validity, if known. Reference to where data collection forms can be found, if not in the protocol	__12________

	18b	Plans to promote participant retention and complete follow-up, including list of any outcome data to be collected for participants who discontinue or deviate from intervention protocols	_n/a__________
Data management	19	Plans for data entry, coding, security, and storage, including any related processes to promote data quality (eg, double data entry; range checks for data values). Reference to where details of data management procedures can be found, if not in the protocol	__12________
Statistical methods	20a	Statistical methods for analysing primary and secondary outcomes. Reference to where other details of the statistical analysis plan can be found, if not in the protocol	__11___________
	20b	Methods for any additional analyses (eg, subgroup and adjusted analyses)	__11 __________
	20c	Definition of analysis population relating to protocol non-adherence (eg, as randomised analysis), and any statistical methods to handle missing data (eg, multiple imputation)	__n/a__________
**Methods: Monitoring**			
Data monitoring	21a	Composition of data monitoring committee (DMC); summary of its role and reporting structure; statement of whether it is independent from the sponsor and competing interests; and reference to where further details about its charter can be found, if not in the protocol. Alternatively, an explanation of why a DMC is not needed	__5___________
	21b	Description of any interim analyses and stopping guidelines, including who will have access to these interim results and make the final decision to terminate the trial	__11___________
Harms	22	Plans for collecting, assessing, reporting, and managing solicited and spontaneously reported adverse events and other unintended effects of trial interventions or trial conduct	___10__________
Auditing	23	Frequency and procedures for auditing trial conduct, if any, and whether the process will be independent from investigators and the sponsor	__n/a_________
**Ethics and dissemination**			
Research ethics approval	24	Plans for seeking research ethics committee/institutional review board (REC/IRB) approval	___17__________

Protocol amendments	25	Plans for communicating important protocol modifications (eg, changes to eligibility criteria, outcomes, analyses) to relevant parties (eg, investigators, REC/IRBs, trial participants, trial registries, journals, regulators)	__n/a_________
Consent or assent	26a	Who will obtain informed consent or assent from potential trial participants or authorised surrogates, and how (see Item 32)	__17____________
	26b	Additional consent provisions for collection and use of participant data and biological specimens in ancillary studies, if applicable	__n/a___________
Confidentiality	27	How personal information about potential and enrolled participants will be collected, shared, and maintained in order to protect confidentiality before, during, and after the trial	___12__________
Declaration of interests	28	Financial and other competing interests for principal investigators for the overall trial and each study site	___17___________
Access to data	29	Statement of who will have access to the final trial dataset, and disclosure of contractual agreements that limit such access for investigators	__17__________
Ancillary and post-trial care	30	Provisions, if any, for ancillary and post-trial care, and for compensation to those who suffer harm from trial participation	___n/a________
Dissemination policy	31a	Plans for investigators and sponsor to communicate trial results to participants, healthcare professionals, the public, and other relevant groups (eg, via publication, reporting in results databases, or other data sharing arrangements), including any publication restrictions	__12__________
	31b	Authorship eligibility guidelines and any intended use of professional writers	___n/a________
	31c	Plans, if any, for granting public access to the full protocol, participant-level dataset, and statistical code	___17___________
**Appendices**			
Informed consent materials	32	Model consent form and other related documentation given to participants and authorised surrogates	__n/a_________
Biological specimens	33	Plans for collection, laboratory evaluation, and storage of biological specimens for genetic or molecular analysis in the current trial and for future use in ancillary studies, if applicable	__n/a_________

For peer review only - http://bmjopen.bmj.com/site/about/guidelines.xhtml

*It is strongly recommended that this checklist be read in conjunction with the SPIRIT 2013 Explanation & Elaboration for important clarification on the items. Amendments to the protocol should be tracked and dated. The SPIRIT checklist is copyrighted by the SPIRIT Group under the Creative Commons "Attribution-NonCommercial-NoDerivs 3.0 Unported" license.

For peer review only

Clinical Centers, Committees, and Resource Centers for the NAITRE Study

Clinical Centers

Centre de Recherche en Sante de Nouna, Nouna, Burkina Faso
Ali Sie (Principal Investigator), Mamadou Bountogo, Mamadou Ouattara, Boubacar Coulibaly, Cheikh Bagagnan, Alphonse Zakane, Lucienne Ouermi, Aristide Ouedraogo, Ouedraogo Andiyam Thierry, Eric Nebie

Centre Hospitalier Universitaire Pédiatrique Charles De Gaulle, Ouagadougou, Burkina Faso
A.M. Napon

Centre Hospitalier Souro Sanou, Bobo Dioulasso, Burkina Faso
Z. Nikièma

Committees

Data and Safety Monitoring Committee:
Allen Hightower (Chair), Amza Abdou, Miriam Laufer, Jacqueline Glover, and Wafaie Fawzi

Resource Centers

Trial Coordinating Center, F.I. Proctor Foundation, University of California, San Francisco, California, USA:
Jessica Brogdon, Catherine Cook, Thuy Doan, William W. Godwin, Jeremy D. Keenan, Elodie Lebas, Thomas M. Lietman (Principal Investigator), Ying Lin, Kieran S. O'Brien, Catherine E. Oldenburg, Travis C. Porco

Heidelberg University, Heidelberg Germany:
Till Bärnighausen

For peer review only

BMJ Open

Neonatal azithromycin administration to prevent infant mortality: study protocol for a randomized controlled trial

Journal:	*BMJ Open*
Manuscript ID	bmjopen-2019-031162.R1
Article Type:	Protocol
Date Submitted by the Author:	23-Jul-2019
Complete List of Authors:	Sie, Ali; Centre de Recherche en Sante de Nouna<br>Bountogo, Mamadou; Centre de Recherche en Sante de Nouna<br>Nebie, Eric; Centre de Recherche en Sante de Nouna<br>Ouattara, Mamadou; Centre de Recherche en Sante de Nouna<br>Coulibaly, Boubacar; Centre de Recherche en Sante de Nouna<br>Bagagnan, Cheik; Centre de Recherche en Sante de Nouna<br>Zabre, Pascal; Centre de Recherche en Sante de Nouna<br>Lebas, Elodie; University of California San Francisco, Francis I Proctor Foundation<br>Brogdon, Jessica; University of California San Francisco, Francis I Proctor Foundation<br>Godwin, William; University of California San Francisco, Francis I Proctor Foundation<br>Lin, Ying; University of California San Francisco, Francis I Proctor Foundation<br>Porco, Travis; University of California, San Francisco, F.I. Proctor Foundation; University of California, San Francisco, Department of Ophthalmology<br>Doan, Thuy; University of California San Francisco, Francis I Proctor Foundation<br>Lietman, Thomas; University of California, San Francisco<br>Oldenburg, Catherine; University of California San Francisco, Francis I Proctor Foundation
**Primary Subject Heading**:	Paediatrics
Secondary Subject Heading:	Global health, Public health, Infectious diseases
Keywords:	azithromycin, neonatal mortality, sub-Saharan Africa, randomized controlled trial, infant mortality

SCHOLARONE™
Manuscripts

Neonatal azithromycin administration to prevent infant mortality: study protocol for a randomized controlled trial

Ali Sié[1], Mamadou Bountogo[1], Eric Nebie[1], Mamadou Ouattara[1], Boubacar Coulibaly[1], Cheik Bagagnan[1], Pascal Zabré[1], Elodie Lebas[2], Jessica M. Brogdon[2], William W. Godwin[2], Ying Lin[2], Travis C. Porco[2,3,4], Thuy Doan[2,3], Thomas M. Lietman[2,3,4], Catherine E. Oldenburg[2,3,4*] for the NAITRE Study Group

[1]Centre de Recherche en Santé de Nouna, Nouna, Burkina Faso
[2]Francis I. Proctor Foundation, University of California, San Francisco, USA
[3]Department of Ophthalmology, University of California, San Francisco, USA
[4]Department of Epidemiology and Biostatistics, University of California, San Francisco, USA

***Author for Correspondence:**
Catherine E. Oldenburg, ScD MPH
513 Parnassus Ave, Room S334
San Francisco, CA, USA
Phone: 415-502-8843
Email: catherine.oldenburg@ucsf.edu

Word Count: 3939
Abstract Word Count: 232
Figures: 2
References: 32

For peer review only

For peer review only - http://bmjopen.bmj.com/site/about/guidelines.xhtml

ABSTRACT

Introduction. Biannual mass azithromycin distribution to children aged 1-59 months has been shown to reduce all-cause mortality. Children under 28 days of age were not treated due to concerns related to infantile hypertrophic pyloric stenosis (IHPS). Here, we report the design of a randomized controlled trial to evaluate the efficacy and safety of administration of a single dose of oral azithromycin during the neonatal period.

Methods and Analysis. The *Nouveaux-nés et Azithromycine: une Innovation dans le Traitement des Enfants* (NAITRE) study is a double-masked randomized placebo-controlled trial designed to evaluate the efficacy of a single dose of azithromycin (20 mg/kg) for the prevention of child mortality. Newborns (*N*=21,712) aged 8 to 27 days weighing at least 2500 g are 1:1 randomized to a single, directly observed, oral dose of azithromycin or matching placebo. Participants are followed weekly for three weeks after treatment to screen for adverse events, including IHPS. The primary outcome is all-cause mortality at the 6-month study visit.

Ethics and Dissemination. This study was approved by the Institutional Review Boards at the University of California, San Francisco in San Francisco, USA (Protocol #18-25027) and the Comité National d'Ethique pour la Recherche in Ouagadougou, Burkina Faso (Protocol #2018-10-123). The findings of this trial will be presented at local, regional, and international meetings and published in open access peer-reviewed journals.

Trial Registration. This study is registered at clinicaltrials.gov (NCT03682653); prospectively registered pre-results.

Key Words. azithromycin; neonatal mortality; sub-Saharan Africa; randomized controlled trial

For peer review only

For peer review only - http://bmjopen.bmj.com/site/about/guidelines.xhtml

Strengths and Limitations of this Study

- This study is a large individually randomized placebo-controlled trial evaluating a single dose of azithromycin compared to a single dose of matching placebo during the neonatal period for the prevention of infant mortality.
- This study will use an identical matching placebo, which will minimize bias between the two arms, and participants, outcome assessors, and investigators are masked to treatment allocation.
- Infants will be carefully monitored for adverse events following treatment administration to screen for infantile hypertrophic pyloric stenosis (IHPS) and other potential adverse events.
- Limitations of the study include the limited number of assessments that can be made with a large sample size and that the study may be underpowered to detect IHPS, given the expected rarity of the condition.

For peer review only

For peer review only - http://bmjopen.bmj.com/site/about/guidelines.xhtml

INTRODUCTION

The MORDOR study demonstrated that mass azithromycin distribution reduces all-cause child mortality relative to placebo in high mortality settings in sub-Saharan Africa.[1,2] The largest effects were seen in the youngest children, with nearly 25% reduction in mortality in children aged one to five months. Children in this age range have the highest risk of mortality, and thus may have the most to gain from child survival interventions. MORDOR treated children as young as one month of age due to increased mortality in this population, but did not treat children before 28 days of life due to concerns related to potential increased risk of infantile hypertrophic pyloric stenosis (IHPS) related to macrolide use.[3,4] No cases of IHPS or increases in vomiting were found in infants aged 1 to 5 months receiving azithromycin in MORDOR relative to placebo.[5]

Globally, reductions in neonatal mortality have been slower than reduction in post-neonatal mortality, and slow progress in reduction in neonatal mortality has affected overall progress in achieving under-five mortality targets.[6,7] The majority of neonatal deaths occur during the first week of life, a majority of which are due to preterm birth and intrapartum-related conditions.[7,8] Neonatal mortality is more likely to be infectious in nature in the late neonatal period (8-27 days), during which time the most common cause of neonatal mortality is sepsis.[7] Post-neonatal mortality (mortality in children aged 28 to 364 days) is higher than in older children, and in sub-Saharan Africa mortality in this age group is largely infectious in nature.[9] Any effect of azithromycin for prevention of child mortality is likely achieved through reductions in infectious mortality.[10] Distribution of azithromycin during early infancy when the risk of infectious mortality is highest may prove to be an efficacious strategy for targeting azithromycin treatment

For peer review only - http://bmjopen.bmj.com/site/about/guidelines.xhtml

for child survival. Here, we describe an individually randomized placebo-controlled double-masked trial designed to establish the efficacy of administration of azithromycin during the neonatal period for the prevention of child mortality. Our central hypothesis is that a single oral dose of azithromycin administered during the neonatal period will reduce all-cause infant mortality, compared to a single dose of placebo.

METHODS/DESIGN

Study design. The *Nouveaux-nés et Azithromycine: une Innovation dans le Traitement des Enfants* (NAITRE; clinicaltrials.gov NCT03682653) study is an individually randomized placebo-controlled trial designed to evaluate the efficacy and safety of administration of a single dose of oral azithromycin during the neonatal period for the prevention of infant mortality (**Figure 1**). Neonates aged 8 to 27 days are randomized in a 1:1 fashion to a single dose of oral azithromycin or matching placebo and followed for 12 months. The primary outcome of the trial is 6-month infant mortality (**Figure 2**). Enrollment commenced in April 2019 and is expected to last until mid-2022.

Objective and hypothesis. The objective of this study is to establish the safety and efficacy of administration of a single dose of oral azithromycin to neonates aged 8 to 27 days for the prevention of infant mortality. We hypothesize that neonates randomized to azithromycin will have significantly lower all-cause mortality by 6 months of age compared to those randomized to placebo.

For peer review only

Study oversight. An independent Data and Safety Monitoring Committee (DSMC) oversees data collection and patient safety in this study. The DSMC contains members with expertise in pediatrics, infectious disease, biostatistics, bioethics, and mass azithromycin distribution. The DSMC meets annually in a face-to-face meeting, including once prior to commencement of the study. Quarterly phone meetings during the implementation of the trial take place to monitor progress and review safety data. In addition to the DSMC, a trial steering committee headquartered in Burkina Faso annually reviews the progress of the meeting. The steering committee meets annually to review trial progress and consists of key stakeholders in policy, child health, and research in Burkina Faso. Serious adverse events are reviewed by two medical monitors, one based in Burkina Faso and one in the United States.

Setting. This study is taking place in 9 regions throughout Burkina Faso. All study sites are within a four-hour drive of a facility with capability of performing pyloromyotomy (Centre Hospitalier Universitaire Pédiatrique Charles de Gaulle in Ouagadougou or Centre Hospitalier Universitaire Souro Sanou in Bobo Dioulasso). Study sites are situated in peri-urban (e.g., outside of Ouagadougou) or rural areas. Pregnancies, births, and newborns will be identified and enrolled at a *Centre de Santé et de Promotion Sociale* (CSPS) in the study area. CSPSs are government nurse-led primary care facilities that offer preventive and curative care to catchment areas covering several rural villages.[11] Study headquarters are at the Centre de Recherche en Santé de Nouna in Nouna, northwestern Burkina Faso. All study personnel underwent didactic and hands-on training in good clinical practice, trial conduct, and trial procedures. Study supervisors and investigators regularly review study data and conduct site visits to ensure adherence to the trial protocol.

For peer review only - http://bmjopen.bmj.com/site/about/guidelines.xhtml

Recruitment. Newborns are recruited through several mechanisms, including identification of pregnant women in their third trimester, facility-based births, key informant or community health worker systems in study areas, and through vaccination days. Pregnant women are informed about the study and asked to contact study staff after delivery if they are interested in their child participating in the trial. Women who give birth in facilities participating in the study are approached prior to discharge about the study and asked to return with their infant in 1 to 2 weeks for eligibility assessment and enrollment. Verbal consent is obtained from pregnant women and women who have recently delivered to collect contact information, follow their pregnancy, and contact them when the child is in the eligible window for enrollment if they are interested in their child participating in the study. Caregivers and newborns are also referred to the study by community health workers and key informants who work in the communities in the catchment areas for participating study clinics. Finally, women who return to health facilities with their infants for vaccination during the neonatal period are approached about the study if their child is in the eligible age range. In this setting, the bacille Calmette-Guérin (BCG) vaccination is typically given in the weeks following birth on a specified "BGC vaccination day", and caregivers attending vaccination days are thus informed about the study. Caregivers of all children in the eligible age range are informed about the study.

Inclusion and exclusion criteria. Newborns are eligible to be enrolled if they are from 8 to 27 days of age, weigh at least 2500 g at the time of enrollment, have no known allergy to azalides, no neonatal jaundice potentially indicating hepatic failure, and are able to feed orally. Children who are too young or too small at initial evaluation can return for a second evaluation for

For peer review only

inclusion in the trial as long as they are in the study's enrollment age range. While lower weight (which may be indicative of younger gestational age, low birthweight, or failure to thrive) children may benefit from the azithromycin intervention, they are excluded from the trial due to mixed evidence of the role of birthweight and gestational age on risk of IHPS, some of which indicates that smaller babies are at increased risk of IHPS.[12] In addition, the family of the newborn must intend to stay in the study area for at least 6 months to minimize loss to follow-up and appropriate consent must be obtained from the caregiver.

Enrollment and baseline survey. After screening and prior to formal enrollment, written informed consent is obtained from the caregiver of all participants. Participants are then enrolled into the study and assigned a study identification number. The baseline assessment includes anthropometry (weight, length, and mid-upper arm circumference measurement), child's birthweight (obtained from the child's health card), timing of initiation of breastfeeding, and pregnancy type (singleton or multiple).

Randomization. Children are randomized in a 1:1 fashion to a single dose of azithromycin or placebo. The randomization list was generated by TCP and WWG in R version 3.3. Treatment letters that correspond to azithromycin or placebo were randomly assigned to study identification numbers. Children are assigned via the study's mobile application to a study identification number and thus a randomization letter. To prevent accidental unmasking, a total of 8 letters are used in the study, with four referring to azithromycin and four referring to placebo. Treatment bottles are labeled with study-specific labels that are identical in appearance with the exception of the treatment letter to facilitate masking.

For peer review only - http://bmjopen.bmj.com/site/about/guidelines.xhtml

Treatment and masking. Treatment is provided as a single oral directly observed dose of azithromycin (approximating 20 mg/kg, weight-based dosing) or matching placebo. Azithromycin for oral suspension is provided in bottles containing azithromycin dehydrate powder equivalent to 1200 mg per bottle. The placebo oral suspension is identical to the azithromycin with the exception of the active ingredient to achieve masking. After children are weighed as part of the baseline anthropometric assessment, the tablet will automatically calculate the dosage of treatment. All study medications, including azithromycin and matching placebo, are donated by Pfizer, Inc.

Allocation concealment. Allocation concealment is achieved via masking and the electronic tablet. With a fully-masked trial, study staff, investigators, and participants are not aware of which assignment they will be given or have received. Furthermore, study staff are not aware of the randomization letters that will be assigned to the next participant until that participant has undergone screening, informed consent, baseline assessment, and randomization. After the baseline visit is complete, the tablet displays the treatment letters corresponding to the medication bottle with which the child will be treated. Viewing treatment letters prior to completion of the baseline form is not possible on the tablet, thus achieving allocation concealment.

Follow-up. All participants complete follow-up visits weekly for three weeks after treatment, and then at 3, 6, and 12 months of age. The weekly adverse event follow-up visits and the 3- and 12-month visits occur via phone call or home visit. The 6-month visit is an in-person visit

For peer review only - http://bmjopen.bmj.com/site/about/guidelines.xhtml

conducted at the CSPS. To minimize loss to follow-up, several forms of contact information are obtained for each participant as well as their location of residence. Community health workers and key informants help facilitate follow-up visits, and home visits are conducted in case participants are not contactable. For the primary outcome, a child's vital status must be known to be considered not lost to follow-up (e.g., the child is known to be alive at 6 months of age or known to have died at or before the 6-month visit). Information is recorded for whether the child was alive, died, had moved, or their status was unknown at each time point.

Primary outcome measurement. The primary outcome is all-cause mortality determined at the 6-month study visit. Vital status is measured via interview with the caregiver or head of household. Children who are living will be evaluated in person at the CSPS or via home visit. Vital status will be recorded in the study's mobile application via tablet.

Secondary outcome measurements.

12-month mortality. Vital status will be measured via caregiver interview determined at 12 months of age. The 12-month study visit will be conducted via phone call or in-person visit.

Neonatal mortality. As a pre-specified secondary outcome, we will assess neonatal mortality (mortality prior to 28 days of age) by study arm. Vital status will be measured in all children at each weekly follow-up visit for three weeks, and as such 28-day mortality will be measured in all children regardless of age of enrollment. Although we do not anticipate that this secondary analysis will be fully powered, it is pre-specified to assess if azithromycin dosing during the neonatal period has a large effect on neonatal mortality.

For peer review only - http://bmjopen.bmj.com/site/about/guidelines.xhtml

Hospitalization. At each study visit, interviewers will be asked if they had sought medical care for their child since they last spoke with the study team, and if so if their child had stayed overnight in a healthcare facility.

Anthropometric outcomes. At the 6-month study visit, anthropometric measurements including weight, length, and mid-upper arm circumference are collected from all children. Weight gain from baseline in grams per kilogram per day (g/kg/day) will be calculated to estimate weight gain velocity by study arm. Weight gain velocity in g/kg/day is a commonly used metric for weight gain, nutritional status over time, and identification of growth deficits in newborns.[13,14] Length gain in mm/day will be calculated by study arm. Weight-for-height Z-score (to measure wasting), height-for-age Z-score (to measure stunting), and weight-for-age Z-score (to measure underweight) will be calculated according to 2006 World Health Organization (WHO) standards.[15]

Cause-specific mortality. A verbal autopsy interview will be undertaken with the caregiver of all children who die during the course of the study using the 2014 WHO verbal autopsy instrument or the neonatal verbal autopsy instrument if the death occurs before 4 weeks of age. Cause of death data will be analyzed using the InterVA algorithm.[16,17]

Adverse events. The primary adverse event under surveillance is IHPS. Active surveillance for IHPS and other adverse events occurs via weekly screening of all enrolled children for three weeks after treatment and at 3 months of age. Although evidence of timing of potentially

For peer review only - http://bmjopen.bmj.com/site/about/guidelines.xhtml

macrolide-related IHPS is limited, previous studies have documented that pyloromyotomy was performed 2-4 weeks following exposure[4], with symptoms developing prior to surgical correction. IHPS development is rare in children older than 6 weeks of age and is not expected to occur after 3 months of age.[4,18] In addition, during the informed consent process the caregiver of all enrolled children is informed of the signs and symptoms of IHPS and asked to contact study staff should the child exhibit abnormal vomiting. All caregivers are given a fact sheet with information about IHPS and its signs and symptoms. Children are enrolled in facilities where they would seek care if necessary, and all healthcare personnel in the facilities are aware of study activities. If a child enrolled in the study is brought to the facility, study staff are informed. If active or passive surveillance identifies a child with projectile or progressive vomiting that occurs consistently after eating (i.e., the child is unable to eat anything without vomiting), the child will be referred to the district hospital for evaluation of clinical symptoms and possible referral to the regional or national hospital for an ultrasound. Any child with projectile vomiting is followed until resolution. If the ultrasound indicates that the pylorus is hypertrophied per Burkinabé national guidelines (>4 mm for pyloric muscle thickness or >15 mm for pyloric length) or the measurements are within normal limits but no food can pass per dynamic ultrasound, the child will be transferred to a national hospital for further evaluation, diagnosis, and pyloromyotomy if IHPS is diagnosed. Children diagnosed with IHPS will be followed at one week and four weeks after surgery to ascertain surgical outcomes, and then according to the study schedule.

In addition to screening for vomiting and IHPS, caregivers are interviewed weekly for three weeks following treatment for adverse events including rash, diarrhea, and fever. Caregivers are

For peer review only

asked if they sought medical care for their child or if the child was hospitalized since the last visit by the study team and if so the indication for seeking treatment or hospitalization. Finally, verbal autopsy is performed for all deaths to ascertain cause of death. The study's DSMC reviewed and approved the adverse event monitoring plan prior to the start of recruitment and reviews all reports of IHPS in real time. Our adverse event monitoring protocol could be modified per DSMC recommendation if, for example, IHPS was more common than anticipated. Although the DSMC reviews each IHPS case in real-time in a masked fashion and study data in aggregate quarterly, they may request IHPS or other safety data by arm if there are concerns related to safety. Each suspected case of IHPS is reviewed by two medical monitors and all suspected cases are followed until resolution.

Interim analysis. One pre-specified interim analysis for the primary outcome is planned when full data are available for one-third of the enrollments or after one full year of enrollment (e.g., 6 months after the last individual), whichever comes first. A *P*-value of <0.001 is the pre-specified stopping rule for efficacy for this interim analysis. Thus the final analysis will be conducted at an alpha of 0.049.

Statistical analysis. The primary analysis will be conducted as a binomial regression model with a complementary log-log link, with inferences based on a Monte Carlo permutation test based on the randomization unit. The complementary log-log link allows for estimation of the relative hazard and can account for differing follow-up time. As a pre-specified subgroup analysis, we will assess effects of age at time of treatment in days using a binomial regression model,

For peer review only

For peer review only - http://bmjopen.bmj.com/site/about/guidelines.xhtml

complementary log-log link, and an interaction for treatment arm by age. All statistical tests for efficacy will be two-sided. A P-value of <0.05 will be considered statistically significant.

Sample size considerations. Assuming a mortality probability of 35 per 1,000 and loss to follow-up of 10%, a sample size of 10,856 per arm (21,712 total) would yield approximately 80% power to detect a 20% decrease in mortality in children randomized to azithromycin compared to placebo.

Data management. Data are collected via smartphone in the field using Survey Solutions (World Bank Group, Washington, DC), a cloud-based platform for electronic data collection. Smartphones are synced in the field to a cloud-based server.

Patient and public involvement. Members of the community, including key informants in the study communities, are involved in identification of births, recruitment of children, and facilitating follow-up visits. Results will be disseminated in collaboration with community leadership in study areas. Patients and the public were not involved in the design or planning of the study.

Ethics and dissemination. Ethical approval was obtained for the NAITRE Study from the Institutional Review Boards at the University of California, San Francisco (Protocol #18-25027) and the Comité National d'Ethique pour la Recherche (National Ethics Committee of Burkina Faso; Protocol #2018-10-123). Written informed consent is obtained from the caregiver of each enrolled child (Supplemental File). Efficacy and safety results of this study will be disseminated

For peer review only - http://bmjopen.bmj.com/site/about/guidelines.xhtml

to community, policy, and scientific stakeholders, including the Ministry of Health in Burkina Faso, the World Health Organization, and relevant non-governmental organizations that implement child health policies. Results will be shared with the scientific community via publication in peer-reviewed journals and presentation at international conferences.

DISCUSSION

Although biannual mass azithromycin distribution has been shown to reduce all-cause child mortality in children aged 1-59 months[1,2], this strategy may not reach the youngest children who may benefit the most. With biannual community distributions, children would first be reached any time between the age of 1 to 7 months, or on average at approximately 4 months of age. Several strategies exist for reliably reaching younger children. For example, quarterly mass treatment would reach children at a younger age on average. Targeted treatment of young children is a strategy that may integrate well with existing health systems and can allow for reliably treated children at specific ages. In Burkina Faso, healthcare for children under 5 is free of charge, and as a result healthcare coverage for children is high. For example, vaccination coverage reportedly exceeded 80% in 2014 in Nouna.[19] If shown to be both efficacious and safe, targeting treatment to the youngest, highest-risk children could be considered via integration with existing well-child visits in the health system.

IHPS is a rare but serious condition, with approximately 2 cases per 1,000 infants in the United States.[4] Surgical treatment is required. However, in many regions of sub-Saharan Africa, infant mortality rates far exceed the risk of IHPS.[20] Prevention of a portion of infant mortality in the highest risk areas may therefore offer substantial benefits for populations. However, the

For peer review only

For peer review only - http://bmjopen.bmj.com/site/about/guidelines.xhtml

associated risks of IHPS as well as the efficacy must be well understood before any such policy could be put into place. In this study, a comprehensive screening and referral protocol is in place to identify and cases of IHPS that arise during the course of the study. We expect that the results of this study will provide evidence of the risks and benefits of treatment of neonates with azithromycin for prevention of child mortality.

To date, existing evidence for the relationship between macrolide use and development of IHPS has been limited to observational studies or small randomized trials of azithromycin for prevention of bronchopulmonary dysplasia in low birthweight infants.[21–23] In a large retrospective cohort study in the United States, among 4,875 infants up to 90 days of life, 8 cases of IHPS occurred, with a higher risk of IHPS among children receiving erythromycin or azithromycin compared to cephalexin.[4] This risk appeared to be higher in children receiving erythromycin compared to azithromycin, and among children under 14 days of age compared to older children. Observational studies are limited by confounding by indication, which could occur if children with conditions necessitating a macrolide have different conditions or are otherwise substantively different from those who receive another antibiotic class or no antibiotics. Existing randomized controlled trials have been small (total N=263 infants randomized), and no cases of IHPS have been detected. Relatively little evidence of the epidemiology of IHPS in sub-Saharan Africa exists[24–26], although some have hypothesized that IHPS is less common in developing country settings compared to high-income settings due to different feeding and medication practices.[26–28] The results of this study are expected to provide

For peer review only - http://bmjopen.bmj.com/site/about/guidelines.xhtml

comprehensive, rigorous evidence of the relationship between azithromycin use and IHPS in neonates, as well as important data on the epidemiology of IHPS in rural West Africa.

Several limitations must be considered in this study. This study was designed under the principle of a large simple trial, due to the large sample size required to be adequately powered for a rare outcome.[29,30] However, careful attention must be paid to each individual in the trial due to the risk of IHPS. The focus is on measuring the most important outcomes (mortality and IHPS), and thus measurement of additional secondary outcomes is limited. For example, the potential for antimicrobial resistance is an important consideration with the use of azithromycin for child health.[31,32] Parallel individual and cluster randomized trials are currently evaluating the effect of azithromycin on selection for macrolide resistance in neonates and older children (clinicaltrials.gov NCT03676751 and NCT03676764). Given the absence of IHPS data from rural sub-Saharan Africa and the expected rarity of the event, the study may be underpowered to detect differences in IHPS between study arms. Stopping rules for safety are based not only on statistical significance, but decisions will be made in conjunction with the DSMC based on case reports arising from the study. The results of this study are expected to be generalizable to neonates in similar West African settings, but results may not be generalizable outside of rural West Africa, where infectious etiology leading to infant mortality may be different. Results may also not be generalizable to low-weight neonates, as they were excluded from the trial. We anticipate that the study will provide the largest sample to date providing evidence of the epidemiology of IHPS in West Africa, as well as the largest trial to date evaluating azithromycin for IHPS.

For peer review only - http://bmjopen.bmj.com/site/about/guidelines.xhtml

CONCLUSIONS

The results of this study are expected to inform policy related to the use of azithromycin for prevention of child mortality by specifically evaluating the role of targeting doses during early infancy when the risk of infectious mortality is highest. This work is anticipated to build on the evidence base arising from the MORDOR study, which demonstrated that biannual mass treatment significantly reduces all-cause child mortality in children aged 1-59 months. If proven to be safe and effective, the evidence arising from this study could be used to develop policies related to neonatal dosing of azithromycin during the neonatal period.

For peer review only

FIGURE LEGENDS

Figure 1. Study flow diagram for study visits and outcome assessments.

Figure 2. SPIRIT schedule of enrollment, interventions, and assessments.

For peer review only

For peer review only - http://bmjopen.bmj.com/site/about/guidelines.xhtml

Contributors. Conceived and designed study: AS, MB, EN, MO, BC, CB, PZ, EL, JMB, WWG, YL, TCP, TD, TML, CEO. Created tables/figures: AS, EL, JMB, CEO. Wrote first draft of manuscript: CEO. Read and critically revised manuscript: AS, MB, EN, MO, BC, CB, PZ, EL, JMB, WWG, YL, TCP, TD, TML, CEO.

Availability of Data and Materials. Upon completion of the trial, de-identified data will be made available in accordance with Bill and Melinda Gates Foundation policies.

Funding. The NAITRE Study is funded by the Bill and Melinda Gates Foundation (OPP1187628). C.E.O and T.D. were supported in part by a Research to Prevention Blindness Career Development Award. The funders played no role in the design, writing, or decision to publish study protocol.

Competing interests. Study medication (azithromycin and placebo) are donated by Pfizer, Inc (New York, NY).

Ethical approval. Ethical approval was obtained for the NAITRE Study from the Institutional Review Boards at the University of California, San Francisco (Protocol #18-25027) and the Comité National d'Ethique pour la Recherche (National Ethics Committee of Burkina Faso; Protocol #2018-10-123).

For peer review only

REFERENCES

1. Keenan JD, Bailey RL, West SK, et al. Mass azithromycin distribution for reducing childhood mortality in sub-Saharan Africa. *N Engl J Med*. 2018;378(17):1583-1592. papers3://publication/uuid/94889A59-49DD-4FAD-BDC0-E1A6B125E22C.
2. Oldenburg CE, Arzika AM, Amza A, et al. Mass azithromycin distribution to prevent childhood mortality: a pooled analysis of cluster randomized trials. *Am J Trop Med Hyg*. 2018;In press:2018.
3. Lund M, Pasternak B, Davidsen RB, et al. Use of macrolides in mother and child and risk of infantile hypertrophic pyloric stenosis: nationwide cohort study. *BMJ*. 2014;348(mar11 8):g1908-g1908. http://www.bmj.com/cgi/doi/10.1136/bmj.g1908.
4. Eberly MD, Eide MB, Thompson JL, Nylund CM. Azithromycin in Early Infancy and Pyloric Stenosis. *Pediatrics*. 2015;135(3):483-488. http://pediatrics.aappublications.org/cgi/doi/10.1542/peds.2014-2026.
5. Oldenburg CE, Arzika AM, Maliki R, et al. Safety of azithromycin in infants under six months of age in Niger : A community randomized trial. 2018:1-11. doi:10.7910/DVN/MQYM5S.Funding
6. Jin Y, Mankadi PM, Rigotti JI. Cause-specific child mortality performance and contributions to all-cause child mortality , and number of child lives saved during the Millennium Development Goals era : a country-level analysis. *Glob Health Action*. 2018;11:1546095. doi:10.1080/16549716.2018.1546095
7. Lawn JE, Blencowe H, Oza S, et al. Every Newborn: progress, priorities, and potential beyond survival. *Lancet*. 2015;384(9938):189-205. doi:10.1016/S0140-6736(14)60496-7
8. Belizan JM, McClure EM, Goudar SS, et al. Neonatal death in low-middle income countries: a global network study. *Am J Perinatol*. 2015;29(8):649-656. doi:10.1055/s-0032-1314885.Neonatal
9. Wang H, Bhutta ZA, Coates MM, et al. Global, regional, national, and selected subnational levels of stillbirths, neonatal, infant, and under-5 mortality, 1980–2015: a systematic analysis for the Global Burden of Disease Study 2015. *Lancet*. 2016;388(10053):1725-1774. http://dx.doi.org/10.1016/S0140-6736(16)31575-6.
10. OBrien KS, Cotter SY, Amza A, et al. Childhood Mortality After Mass Distribution of Azithromycin. *Pediatr Infect Dis J*. 2018;37(11):1082-1086. http://insights.ovid.com/crossref?an=00006454-900000000-96705.
11. Koulidiati J-L, Nesbitt RC, Ouedraogo N, et al. Measuring effective coverage of curative child health services in rural Burkina Faso: a cross-sectional study. *BMJ Open*. 2018;8(5):e020423-10. http://bmjopen.bmj.com/lookup/doi/10.1136/bmjopen-2017-020423.
12. Ranells JD, Carver JD, Kirby RS. Infantile Hypertrophic Pyloric Stenosis: Epidemiology, Genetics, and Clinical Update. *Adv Pediatr*. 2011;58(1):195-206. http://dx.doi.org/10.1016/j.yapd.2011.03.005.
13. Olusanya BO, Renner JK. Predictors of growth velocity in early infancy in a resource-poor setting. *Early Hum Dev*. 2011;87(10):647-652. doi:10.1016/j.earlhumdev.2011.05.002
14. Nobre RG, de Azevedo DV, de Almeida PC, de Almeida NMGS, Feitosa FE de L. Weight-Gain Velocity in Newborn Infants Managed with the Kangaroo Method and Associated Variables. *Matern Child Health J*. 2017;21(1):128-135. doi:10.1007/s10995-

For peer review only

016-2101-2
15. WHO Multicentre Growth Reference Study Group. WHO Child Growth Standards: Length/height-for-age, weight-for-age, weight-for-length, weight-for-height and body mass index-for-age: Methods and development. *Geneva World Heal Organ*. 2006:312.
16. Byass P, Herbst K, Fottrell E, et al. Comparing verbal autopsy cause of death findings as determined by physician coding and probabilistic modeling: a public health analysis of 54 000 deaths in Africa and Asia. *J Glob Health*. 2015;5:1-9. papers3://publication/doi/10.7189/jogh.05.010402.
17. Yé M, Diboulo E, Niamba L, et al. An improved method for physician-certified verbal autopsy reduces the rate of discrepancy: experiences in the Nouna Health and Demographic Surveillance Site (NHDSS), Burkina Faso. *Popul Health Metr*. 2011;9(1):34. http://www.pophealthmetrics.com/content/9/1/34.
18. Hernanz-schulman M. Infantile Hypertrophic Pyloric Stenosis. *Radiology*. 2003;1(2):319-331.
19. Kagoné M, Yé M, Nébié E, et al. Vaccination coverage and factors associated with adherence to the vaccination schedule in young children of a rural area in Burkina Faso. *Glob Health Action*. 2017;10(1):1399749. doi:10.1080/16549716.2017.1399749
20. Golding N, Burstein R, Longbottom J, et al. Mapping under-5 and neonatal mortality in Africa, 2000–15: a baseline analysis for the Sustainable Development Goals. *Lancet*. 2017;390(10108):2171-2182. http://dx.doi.org/10.1016/S0140-6736(17)31758-0.
21. Smith C, Egunsola O, Choonara I, Kotecha S, Jacqz-Aigrain E, Sammons H. Use and safety of azithromycin in neonates: a systematic review. *BMJ Open*. 2015;5(12):e008194-8. http://bmjopen.bmj.com/lookup/doi/10.1136/bmjopen-2015-008194.
22. Ballard HO, Anstead MI, Shook LA. Azithromycin in the extremely low birth weight infant for the prevention of Bronchopulmonary Dysplasia: a pilot study. *Respir Res*. 2007;8(1):1793-1799. http://respiratory-research.biomedcentral.com/articles/10.1186/1465-9921-8-41.
23. Ballard HO, Shook LA, Bernard P, et al. Use of azithromycin for the prevention of bronchopulmonary dysplasia in preterm infants: a randomized, double-blind, placebo controlled trial. *Pediatr Pulmonol*. 2010;46(2):111-118. http://doi.wiley.com/10.1002/ppul.21352.
24. Chalya PL, Manyama M, Kayange NM, Mabula JB, Massenga A. Infantile hypertrophic pyloric stenosis at a tertiary care hospital in Tanzania: a surgical experience with 102 patients over a 5-year period. *BMC Res Notes*. November 2015:1-6. papers3://publication/doi/10.1186/s13104-015-1660-4.
25. Tadesse A, Gadisa A. Infantile hypertrophic pyloric stenosis: A retrospective study from a tertiary hospital in Ethiopia. *East Cent Afr J Surg*. 2014;19(1):120-124. https://www.ajol.info/index.php/ecajs/article/view/102528/92798.
26. Osifo DO, Evbuomwan I. Does Exclusive Breastfeeding Confer Protection Against Infantile Hypertrophic Pyloric Stenosis? A 30-year Experience in Benin City, Nigeria. *J Trop Pediatr*. 2008;55(2):132-134. https://academic.oup.com/tropej/article-lookup/doi/10.1093/tropej/fmn094.
27. Zhu J, Zhu T, Lin Z, Qu Y, Mu D. Perinatal risk factors for infantile hypertrophic pyloric stenosis: A meta-analysis. *J Pediatr Surg*. 2017;52(9):1389-1397. http://dx.doi.org/10.1016/j.jpedsurg.2017.02.017.
28. Wayne C, Hung J-HC, Chan E, Sedgwick I, Bass J, Nasr A. Formula-feeding and

For peer review only - http://bmjopen.bmj.com/site/about/guidelines.xhtml

hypertrophic pyloric stenosis: is there an association? A case–control study. *J Pediatr Surg*. 2016;51(5):779-782. http://dx.doi.org/10.1016/j.jpedsurg.2016.02.021.

29. Yusuf S, Collins R, Peto R. Why do we need some large, simple trials? *Stat Med*. 1984;3:409-420. https://www.readcube.com/articles/10.1002/sim.4780030421?no_additional_access=1&tracking_referrer=onlinelibrary.wiley.com&purchase_referrer=www.google.com&publisher=wiley&access_api=1&parent_url=https:%252F%252Fonlinelibrary.wiley.com%252Fdoi%252Fepdf%252F10.1002%252.
30. Peto R, Collins R, Gray R. Large-scale randomized evidence: large, simple trials and overviews of trials. *J Clin Epidemiol*. 1995;48(1):23-40.
31. O'Brien K, Emerson P, Hooper PJ, et al. Antimicrobial resistance following mass azithromycin distribution for trachoma: a systematic review. *Lancet Infect Dis*. 2018;S1437-3099(18):30444-4. papers3://publication/uuid/DF60F97D-6D3B-4FA4-880C-055146B4B65A.
32. Doan T, Arzika AM, Hinterwirth A, et al. Macrolide Resistance in MORDOR I — A Cluster-Randomized Trial in Niger. *N Engl J Med*. 2019;380(23):2271-2273. doi:10.1056/nejmc1901535

For peer review only

Clinical Centers, Committees, and Resource Centers for the NAITRE Study

Clinical Centers

Centre de Recherche en Sante de Nouna, Nouna, Burkina Faso
Ali Sie (Principal Investigator), Mamadou Bountogo, Mamadou Ouattara, Boubacar Coulibaly, Cheik Bagagnan, Alphonse Zakane, Guillaume Compaoré, Valentin Boudo, Lucienne Ouermi, Aristide Ouedraogo, Ouedraogo Andiyam Thierry, Eric Nebie

Centre Hospitalier Universitaire Pédiatrique Charles De Gaulle, Ouagadougou, Burkina Faso
A.M. Napon

Centre Hospitalier Souro Sanou, Bobo Dioulasso, Burkina Faso
Z. Nikièma

Committees

Data and Safety Monitoring Committee:
Allen Hightower (Chair), Amza Abdou, Miriam Laufer, Jacqueline Glover, and Wafaie Fawzi

Resource Centers

Trial Coordinating Center, F.I. Proctor Foundation, University of California, San Francisco, California, USA:
Jessica Brogdon, Catherine Cook, Thuy Doan, William W. Godwin, Jeremy D. Keenan, Elodie Lebas, Thomas M. Lietman (Principal Investigator), Ying Lin, Kieran S. O'Brien, Catherine E. Oldenburg (Principal Investigator), Travis C. Porco

Heidelberg University, Heidelberg Germany:
Till Bärnighausen

For peer review only

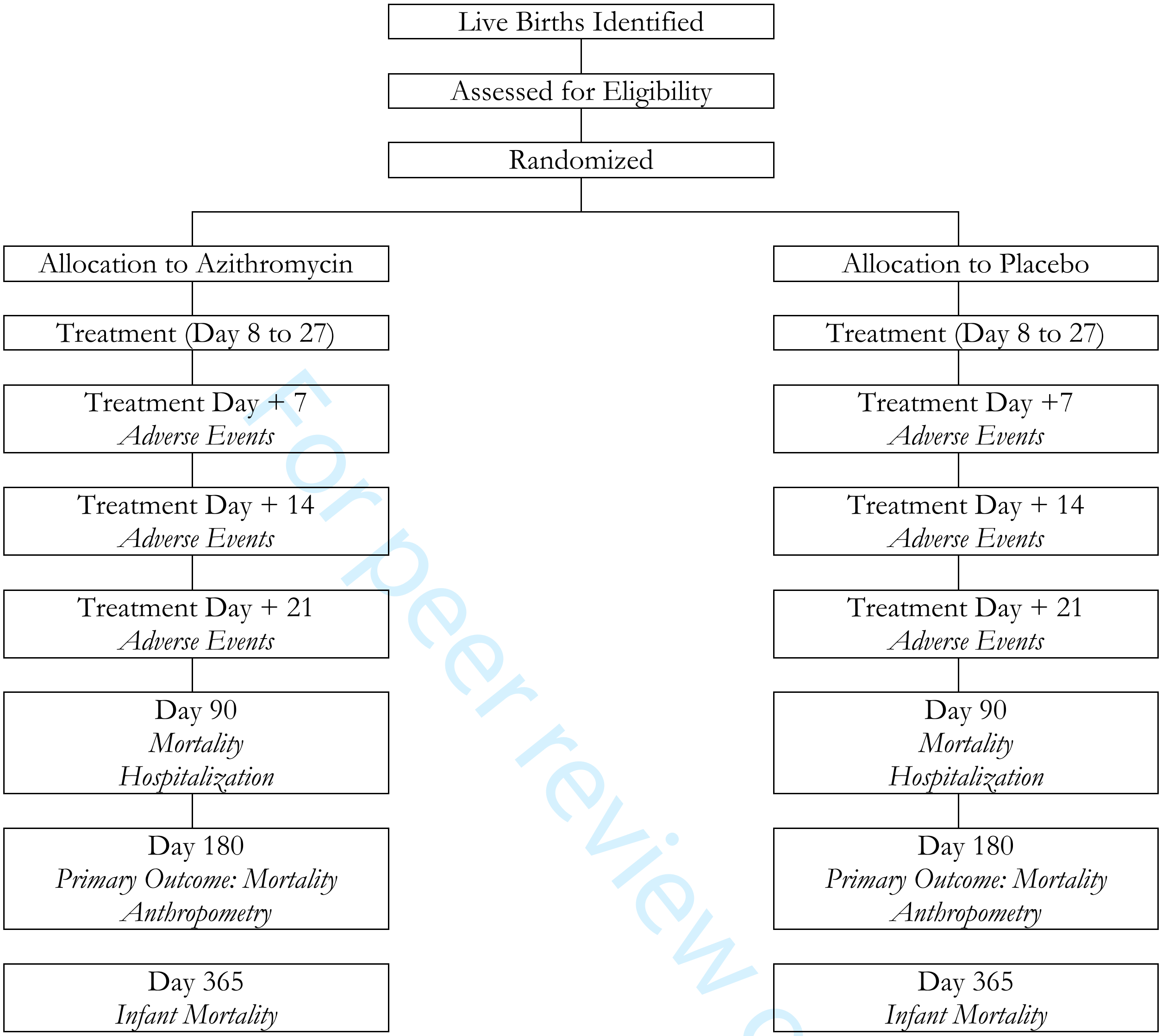

For peer review only

	STUDY PERIOD					
	Enrolment	Allocation	Post-allocation			Close-out
**TIMEPOINT**		*0*	*Weeks 1, 2, 3*	*3 months*	*6 months*	*12 months*
**ENROLMENT:**						
**Eligibility screen**	X					
**Informed consent**	X					
**Allocation**		X				
**INTERVENTIONS:**						
**Treatment (oral azithromycin or placebo)**		X				
**ASSESSMENTS:**						
**Baseline questionnaire**	X					
**Anthropometry**	X				X	
**Adverse events**			X	X		
**Vital status**			X	X	X	X

UNIVERSITY OF CALIFORNIA, SAN FRANCISCO CENTRE DE RECHERCHE EN SANTE DE NOUNA PARENTAL CONSENT TO PARTICIPATE IN A RESEARCH STUDY

This is a medical research study. This study will be explained to you by a staff member from the Centre de Recherche en Santé de Nouna (CRSN). The principal investigator, Dr. Ali Sié from the CRSN, tel: [redacted] and the other principal investigator for this study, Dr Thomas Lietman from the university of California in San Francisco, are available by phone to answer your questions at [redacted].

Medical research studies include only people who choose to take part. Take your time to make your decision about having your child participating in the study. You may discuss your decision with your family and friends. If you have any questions, you may ask your study doctors or the personnel of the CRSN in Nouna.

Why is this study being done?

The purpose of this study is to reduce the childhood mortality during the neonatal period (the first month of life)

How many children will take part in this study?

About 21,000 children will take part in this study.

What will happen if my child takes part in this research study?

If you decide that your child will take part in this study, we will give your child a single oral dose of Azithromycin or placebo between the 8th and the 27th day of life.

Your child might receive Azithromycin, but s/he might also receive a placebo. The placebo will look and taste the same as the azithromycin but will not contain any active medication.

The treatment assignment will be randomly decided by chance, similar to a lottery, with an equal chance of the infant receiving either azithromycin or placebo. The placebo will look and taste the same as Azithromycin but will not contain any active medicine.

After the treatment has been administered we will come to your house 6 times and ask you a few question about your child and his/her health status. For example we will ask you if your child was sick since the last time we saw him/her, we will also ask if your child still lives with you. We will come to your house 7, 14, 28, 90, 180, and 365 days after the treatment day.

Before the treatment administration and when your child is approximatively 6 months old we will measure your child's length, weight and Middle Upper Arm circumference (the size of the contour of your child's arm).

For peer review only

How long will my child be in the study?

The entire time your child will be in the study is approximatively 1 hour and 55 minutes over a period of 1 year.

- The single administration of the treatment will take approximatively 5 minutes.
- Each follow up visit after your child has been treated will take approximatively 15 minutes.
- The anthropometric measurements will take approximatively 10 minutes each time (20 minutes total)

Can my child stop being in the study?

Yes. You can decide to stop at any time. Tell the study doctor or the personnel at the CRSN if you are thinking about stopping or decide to stop. He or she will tell you how to stop your child's participation safely.

What side effects or risks can my child expect from being in the study?

Your child may have side effects due to the treatment.

The most common side effects with this kind of antibiotics are: diarrhea, abdominal pain, vomiting or rash. Every child taking part in this study will be carefully watched for any side effects.

Taking the treatment might also increase the risk of your child developing infantile hypertrophic pyloric stenosis (IHPS). IHPS is when the opening between the stomach and small intestines thickens, if this happens your child will vomit and won't be able to feed properly. The only way to treat IHPS is to perform a surgery that will widen the opening between the stomach and the small intestines. This is an uncommon condition and your child will be monitored very closely for any early signs of IHPS. If your child develops symptoms that might be related to this condition s/he will be seen immediately by a pediatrician for further evaluation.

Your child might also be allergic to the study drug, the most common allergic reactions to this kind of treatment are: rash, itching or dizziness. The serious allergic reactions such as shortness of breath or swelling of the face/tongue/lips are rare and will be monitored closely by our team.

Your child might experience some discomfort during the anthropometric measurements but the risk of hurting your child is minimal. We will take all the precautions to not hurt your child during this procedure and only medical trained staff will perform the measurements.

You should talk to a staff member of the CRSN if your child experiences any side effect.

For peer review only - http://bmjopen.bmj.com/site/about/guidelines.xhtml

Are there benefits to taking part in the study?

If your child is suffering from an undiagnosed infectious disease, s/he might directly benefit from the receipt of azithromycin. Not all children will benefit from being in the study and not all the children will receive treatment by being in the study. The children who receive placebo will not receive any benefit or treatment.

In a recent study done in Niger, Tanzania and Malawi, the administration of the azithromycin reduced by 25% the mortality rate in children aged 1 to 6 months.

What other choices do I have if my child does not take part in this study?

You can decide to take part in this study or not take part in this study. Your participation is voluntary. It is your choice whether or not your child will be part of this study.

Nothing will happen to you or your child if you decide that your child won't take part in this study.

How will information about my child be kept confidential?

We will keep all of your child's information confidential. Some entities might be able to access study records, in particular, the University of California at San Francisco Institutional Review Board, the Centre de Recherche en santé de Nouna, and the US Office for Human Research Protections.

There always exists the risk of loss/breach of confidentiality of study data, but that we will take measures to prevent that from happening. We will not use your child's name for anything, except to make sure they are offered treatment when we come back.

Who can answer my questions about the study?

If you have any questions about the study or your rights as a participant to someone other than the researchers or if you have any concerns about the study, please contact the Chair of the Health Research Ethics Board (HREB) Pr. Seni Kouanda 09 P Boîte 7009 Ouagadougou 09, tel: +226 25 36 6674 mobile: +226 70 261462 or the President of the NSRF Institutional Ethics Committee: Mr Zoumbara Jean Désiré, tel: +22670716530, P Box 02, Nouna.

This is a medical research study. This study will be explained to you by a staff member of the Centre de Recherche en Santé de Nouna (CRSN). Dr Ali Sié, NSRF Principal Investigator, telephone: [redacted] is available to answer your questions, Dr Mamadou Bountogo, NSRF Coordinator, telephone:

For peer review only - http://bmjopen.bmj.com/site/about/guidelines.xhtml

[redacted] and the other Principal Investigator, Dr Thomas Lietman of the University of California at San Francisco, is also available to answer your questions by telephone: [redacted].

For peer review only

For peer review only - http://bmjopen.bmj.com/site/about/guidelines.xhtml

CONSENT

I have read the informed consent form, or it has been read to me. I fully understand the above information. I had the opportunity to ask questions, and I got satisfactory answers. I voluntarily consent for my child to participate in this research. I understand that I have the right to decline to my child's participation or to withdraw at any time without penalty or loss of benefits to which I am otherwise entitled.

____________ ____

Date Participant's Signature or fingerprint *(in case participant is illiterate)*

____________ ____

Date Participant's Witness Signature *(in case participant is illiterate)*

____________ ____

Date Person Obtaining Consent

For peer review only

For peer review only - http://bmjopen.bmj.com/site/about/guidelines.xhtml

STANDARD PROTOCOL ITEMS: RECOMMENDATIONS FOR INTERVENTIONAL TRIALS

SPIRIT 2013 Checklist: Recommended items to address in a clinical trial protocol and related documents*

Section/item	Item No	Description	Addressed on page number
**Administrative information**			
Title	1	Descriptive title identifying the study design, population, interventions, and, if applicable, trial acronym	________1______
Trial registration	2a	Trial identifier and registry name. If not yet registered, name of intended registry	________2______
	2b	All items from the World Health Organization Trial Registration Data Set	_____________
Protocol version	3	Date and version identifier	______n/a______
Funding	4	Sources and types of financial, material, and other support	________17______
Roles and responsibilities	5a	Names, affiliations, and roles of protocol contributors	________1, 17____
	5b	Name and contact information for the trial sponsor	________17______
	5c	Role of study sponsor and funders, if any, in study design; collection, management, analysis, and interpretation of data; writing of the report; and the decision to submit the report for publication, including whether they will have ultimate authority over any of these activities	_________17_____
	5d	Composition, roles, and responsibilities of the coordinating centre, steering committee, endpoint adjudication committee, data management team, and other individuals or groups overseeing the trial, if applicable (see Item 21a for data monitoring committee)	______5______

For peer review only

Introduction

Background and rationale	6a	Description of research question and justification for undertaking the trial, including summary of relevant studies (published and unpublished) examining benefits and harms for each intervention	_____3-4_______
	6b	Explanation for choice of comparators	____4_________
Objectives	7	Specific objectives or hypotheses	_____4________
Trial design	8	Description of trial design including type of trial (eg, parallel group, crossover, factorial, single group), allocation ratio, and framework (eg, superiority, equivalence, noninferiority, exploratory)	_____3-4_______

Methods: Participants, interventions, and outcomes

Study setting	9	Description of study settings (eg, community clinic, academic hospital) and list of countries where data will be collected. Reference to where list of study sites can be obtained	______5_______
Eligibility criteria	10	Inclusion and exclusion criteria for participants. If applicable, eligibility criteria for study centres and individuals who will perform the interventions (eg, surgeons, psychotherapists)	_____6________
Interventions	11a	Interventions for each group with sufficient detail to allow replication, including how and when they will be administered	_____7_____
	11b	Criteria for discontinuing or modifying allocated interventions for a given trial participant (eg, drug dose change in response to harms, participant request, or improving/worsening disease)	_____n/a_______
	11c	Strategies to improve adherence to intervention protocols, and any procedures for monitoring adherence (eg, drug tablet return, laboratory tests)	______n/a______
	11d	Relevant concomitant care and interventions that are permitted or prohibited during the trial	_____n/a______
Outcomes	12	Primary, secondary, and other outcomes, including the specific measurement variable (eg, systolic blood pressure), analysis metric (eg, change from baseline, final value, time to event), method of aggregation (eg, median, proportion), and time point for each outcome. Explanation of the clinical relevance of chosen efficacy and harm outcomes is strongly recommended	___8-9_______

For peer review only - http://bmjopen.bmj.com/site/about/guidelines.xhtml

Participant timeline	13	Time schedule of enrolment, interventions (including any run-ins and washouts), assessments, and visits for participants. A schematic diagram is highly recommended (see Figure)	__8; Figure 2____
Sample size	14	Estimated number of participants needed to achieve study objectives and how it was determined, including clinical and statistical assumptions supporting any sample size calculations	___11__________
Recruitment	15	Strategies for achieving adequate participant enrolment to reach target sample size	___6__________
**Methods: Assignment of interventions (for controlled trials)**			
Allocation:			
Sequence generation	16a	Method of generating the allocation sequence (eg, computer-generated random numbers), and list of any factors for stratification. To reduce predictability of a random sequence, details of any planned restriction (eg, blocking) should be provided in a separate document that is unavailable to those who enrol participants or assign interventions	___7__________
Allocation concealment mechanism	16b	Mechanism of implementing the allocation sequence (eg, central telephone; sequentially numbered, opaque, sealed envelopes), describing any steps to conceal the sequence until interventions are assigned	___8__________
Implementation	16c	Who will generate the allocation sequence, who will enrol participants, and who will assign participants to interventions	___7-8________
Blinding (masking)	17a	Who will be blinded after assignment to interventions (eg, trial participants, care providers, outcome assessors, data analysts), and how	___7________
	17b	If blinded, circumstances under which unblinding is permissible, and procedure for revealing a participant's allocated intervention during the trial	__7_________
**Methods: Data collection, management, and analysis**			
Data collection methods	18a	Plans for assessment and collection of outcome, baseline, and other trial data, including any related processes to promote data quality (eg, duplicate measurements, training of assessors) and a description of study instruments (eg, questionnaires, laboratory tests) along with their reliability and validity, if known. Reference to where data collection forms can be found, if not in the protocol	__12________

	18b	Plans to promote participant retention and complete follow-up, including list of any outcome data to be collected for participants who discontinue or deviate from intervention protocols	_n/a__________
Data management	19	Plans for data entry, coding, security, and storage, including any related processes to promote data quality (eg, double data entry; range checks for data values). Reference to where details of data management procedures can be found, if not in the protocol	__12________
Statistical methods	20a	Statistical methods for analysing primary and secondary outcomes. Reference to where other details of the statistical analysis plan can be found, if not in the protocol	__11___________
	20b	Methods for any additional analyses (eg, subgroup and adjusted analyses)	__11 __________
	20c	Definition of analysis population relating to protocol non-adherence (eg, as randomised analysis), and any statistical methods to handle missing data (eg, multiple imputation)	__n/a__________
**Methods: Monitoring**			
Data monitoring	21a	Composition of data monitoring committee (DMC); summary of its role and reporting structure; statement of whether it is independent from the sponsor and competing interests; and reference to where further details about its charter can be found, if not in the protocol. Alternatively, an explanation of why a DMC is not needed	__5___________
	21b	Description of any interim analyses and stopping guidelines, including who will have access to these interim results and make the final decision to terminate the trial	__11___________
Harms	22	Plans for collecting, assessing, reporting, and managing solicited and spontaneously reported adverse events and other unintended effects of trial interventions or trial conduct	___10__________
Auditing	23	Frequency and procedures for auditing trial conduct, if any, and whether the process will be independent from investigators and the sponsor	__n/a_________
**Ethics and dissemination**			
Research ethics approval	24	Plans for seeking research ethics committee/institutional review board (REC/IRB) approval	___17__________

Protocol amendments	25	Plans for communicating important protocol modifications (eg, changes to eligibility criteria, outcomes, analyses) to relevant parties (eg, investigators, REC/IRBs, trial participants, trial registries, journals, regulators)	__n/a_________
Consent or assent	26a	Who will obtain informed consent or assent from potential trial participants or authorised surrogates, and how (see Item 32)	__17____________
	26b	Additional consent provisions for collection and use of participant data and biological specimens in ancillary studies, if applicable	__n/a___________
Confidentiality	27	How personal information about potential and enrolled participants will be collected, shared, and maintained in order to protect confidentiality before, during, and after the trial	___12__________
Declaration of interests	28	Financial and other competing interests for principal investigators for the overall trial and each study site	___17___________
Access to data	29	Statement of who will have access to the final trial dataset, and disclosure of contractual agreements that limit such access for investigators	__17__________
Ancillary and post-trial care	30	Provisions, if any, for ancillary and post-trial care, and for compensation to those who suffer harm from trial participation	___n/a________
Dissemination policy	31a	Plans for investigators and sponsor to communicate trial results to participants, healthcare professionals, the public, and other relevant groups (eg, via publication, reporting in results databases, or other data sharing arrangements), including any publication restrictions	__12__________
	31b	Authorship eligibility guidelines and any intended use of professional writers	___n/a________
	31c	Plans, if any, for granting public access to the full protocol, participant-level dataset, and statistical code	___17___________
**Appendices**			
Informed consent materials	32	Model consent form and other related documentation given to participants and authorised surrogates	__n/a_________
Biological specimens	33	Plans for collection, laboratory evaluation, and storage of biological specimens for genetic or molecular analysis in the current trial and for future use in ancillary studies, if applicable	__n/a_________

For peer review only - http://bmjopen.bmj.com/site/about/guidelines.xhtml

*It is strongly recommended that this checklist be read in conjunction with the SPIRIT 2013 Explanation & Elaboration for important clarification on the items. Amendments to the protocol should be tracked and dated. The SPIRIT checklist is copyrighted by the SPIRIT Group under the Creative Commons "Attribution-NonCommercial-NoDerivs 3.0 Unported" license.

For peer review only
